# Review of Breathable Electronics

**DOI:** 10.3390/ma19204412

**Published:** 2026-10-20

**Authors:** Jialin Fan, Jiamiao Li, Shanmei Li, Ruoying Hong, Jianfeng Ma, Bowen Lei, Peiyan Dong, Chenzi Fan, Qiyuan Xiao, Jianhua Zhou, Yancong Qiao

**Affiliations:** 1 School of Biomedical Engineering, Shenzhen Campus of Sun Yat-sen University, No. 66, Gongchang Road, Guangming District, Shenzhen 518107, China; 2 Key Laboratory of Sensing Technology and Biomedical Instruments of Guangdong Province, School of Biomedical Engineering, Sun Yat-sen University, Guangzhou 510275, China

**Keywords:** breathable electronics, epidermal electronics, flexible sensors, electronic skin, health monitoring

## Abstract

When applied to the human body, flexible wearable devices must not only address issues of mechanical fit and biocompatibility, but also overcome key bottlenecks, such as skin irritation, signal drift, and poor stability caused by the insufficient breathability of traditional flexible electronics. Breathable electronics have emerged in response to these challenges. Characterized by high breathability, low irritation and conformable adhesion, they are dedicated to resolving problems, such as skin irritation and poor long-term signal stability caused by the lack of breathability in traditional flexible electronics. Therefore, this review first systematically outlines the implementation strategies for breathable electronics, including the preparation of stretchable substrates, the incorporation of breathable microstructures, and strategies for forming conductive pathways. Subsequently, the principles, materials, and performance characteristics of key discrete components are introduced, such as interconnect circuits and energy devices. Cutting-edge applications of breathable electronics in fields such as health monitoring and electronic skin are summarized. The construction of fully functional systems is further explored, which is primarily in the form of fabric-integrated and patch-based devices, through system integration strategies. Subsequently, the long-term failure mechanisms in practical deployment, including spanning sweat corrosion, mechanical fatigue, washing damage, and environmental aging, are critically examined, alongside corresponding material, structural, and algorithmic reliability enhancement strategies. Finally, the challenges facing breathable electronics technology in terms of system integration and long-term stability are analyzed, and the potential for future development through interdisciplinary convergence is proposed.

## 1. Introduction

As the global population continues to age and the burden of managing chronic diseases grows, the modern healthcare model is undergoing a profound transformation, namely shifting from a passive, in-hospital clinical care model to a proactive, home-based, personalized, and round-the-clock health monitoring approach [1]. As the core hardware platform for this transformation, flexible wearable electronics can non-invasively and in real time collect multidimensional physiological data, such as heart rate, blood oxygen levels, and sweat, thereby breaking down the barriers that traditionally confined medical devices to hospital settings and enabling early disease screening and long-term home monitoring of chronic conditions [2,3,4,5].

Currently, flexible epidermal electronics have made significant progress in mechanical adaptation: through designs such as elastic substrates and flexural microstructures, devices can match the elastic modulus of human soft tissues, enabling them to conform closely to curved skin surfaces for signal acquisition [6,7,8]. However, these flexible devices have a significant limitation: insufficient breathability. Most traditional flexible electronic devices rely on dense elastomers (such as polydimethylsiloxane (PDMS) and polyurethane (PU) films) as encapsulation substrates [9,10]. Although these materials possess excellent mechanical properties, their extremely low water vapor permeability (WVP) causes sweat and heat to accumulate at the skin–device interface, leading to a skin occlusion effect. This effect not only causes skin inflammation and discomfort during long-term wear, but also leads to severe signal artifacts and baseline drift due to fluctuations in skin-interface impedance, thereby limiting the practical application of flexible electronic devices in long-term continuous monitoring scenarios [11,12,13].

To address these challenges, breathable electronics technology has emerged as a design paradigm for integrating electronic functionality with efficient gas and moisture transport. In this review, breathable electronics are defined as electronic devices that maintain their electrical, sensing, and signal-transmission functions while allowing the transport of water vapor, air, and heat between the skin and the external environment. Based on a core design philosophy centered on permeable structures, such as porous nanofibers, microperforations, and biomimetic layered structures, breathable electronic devices facilitate moisture dissipation and reduce skin occlusion while preserving electrical functionality, sensing performance, circuit transmission, and mechanical deformability [1,14,15,16]. By regulating breathable interfaces, this technology maintains a stable skin-electrode contact environment, ensuring long-term, high-fidelity acquisition of complex physiological signals and laying a solid foundation for a new generation of skin-integrated health monitoring platforms [17,18].

The breathability of electronic devices can be evaluated using several complementary parameters, including the water vapor transmission rate (WVTR), air permeability, and porosity. WVTR reflects the rate of water-vapor transport through the device, air permeability characterizes gas flow through the material under a defined pressure difference, and porosity describes the fraction of void space that facilitates gas and moisture transport. In contrast to conventional flexible electronics based on dense, continuous elastomeric films, breathable electronics incorporate interconnected porous, fibrous, or micropatterned pathways to promote water-vapor and air exchange while maintaining electrical and mechanical functions. Therefore, the breathability of an electronic device depends not only on the material itself but also on its morphology and structural design.

Although the field of breathable electronics has garnered significant attention in recent years, most previous research reviews have been limited to specific material systems or single structural design strategies, lacking a comprehensive, systematic overview spanning the entire chain: from substrate design, conductive modification, functional components, and system integration to actual medical applications [19,20]. In contrast, this review adopts a comprehensive, integrated approach centered on the material-device-system framework. Its core lies in bridging three major research dimensions: intrinsic material modification, microstructural topology design, and heterogeneous compatibility between hardware and software. Thereby systematically analyzing the entire process of breathable electronics, from fundamental fabrication to wearable implementation, and providing a more comprehensive reflection of the complete development trajectory of this interdisciplinary field.

**Figure 1:**
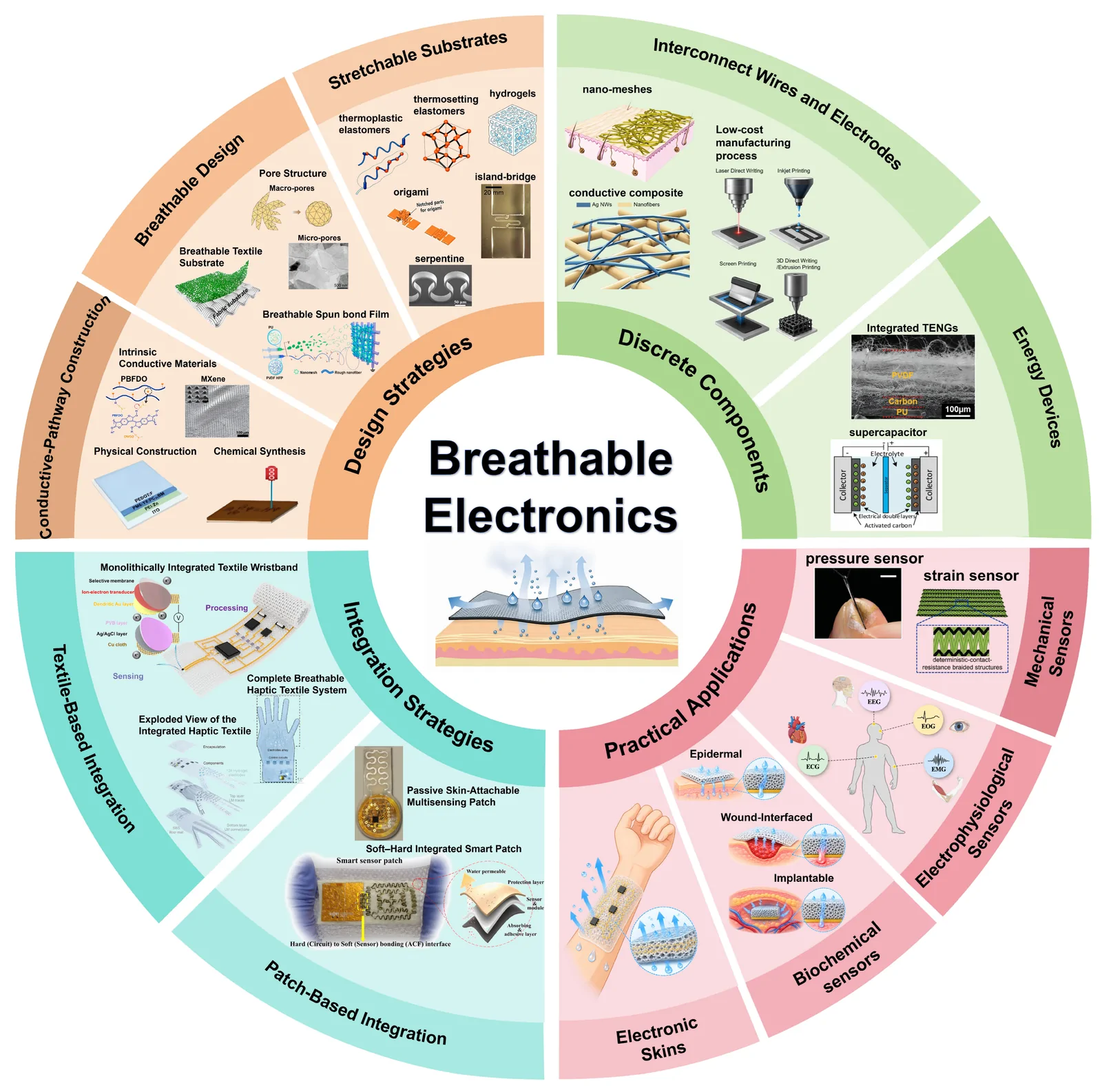
Schematic of the overall contents of this review. Stretchable Substrates. Reproduced with permission [21]. Copyright 2019, American Chemical Society. Reproduced with permission [22]. Copyright 2020, WILEY. Reproduced with permission [23]. Copyright 2024, WILEY. Breathable Design. Reproduced with permission [24]. Copyright 2026, Springer Nature. Reproduced with permission [25]. Copyright 2024, WILEY. Reproduced with permission [26]. Copyright 2022, American Association for the Advancement of Science. Reproduced with permission [27]. Copyright 2023, American Chemical Society. Conductive-Pathway Construction. Reproduced with permission [28]. Copyright 2026, WILEY. Reproduced with permission [29]. Copyright 2022, American Association for the Advancement of Science. Reproduced with permission [30]. Copyright 2022, WILEY. Reproduced with permission [31]. Copyright 2018, American Association for the Advancement of Science. Interconnect Wires and Electrodes. Reproduced with permission [32]. Copyright 2017, Springer Nature. Reproduced with permission [33]. Copyright 2018, American Chemical Society. Energy Devices. Reproduced with permission [34]. Copyright 2020, WILEY. Reproduced with permission [35]. Copyright 2022, MDPI. Mechanical Sensors. Reproduced with permission [36]. Copyright 2020, American Association for the Advancement of Science. Reproduced with permission [37]. Copyright 2022, WILEY. Textile-Based Integration. Reproduced with permission [38]. Copyright 2022, American Association for the Advancement of Science. Reproduced with permission [39]. Copyright 2024, American Association for the Advancement of Science. Patch-Based Integration. Reproduced with permission [40]. Available under a CC-BY 4.0 license. Reproduced with permission [41]. Available under a CC-BY 4.0 license.

This review focuses on the research-to-application pathway for breathable electronics, exploring the subject layer by layer across all dimensions: from the design of underlying material structures and the fabrication of core functional devices to the integration of complete systems and their practical physiological applications. The overall contents and organization of this review are summarized in Figure 1. The paper is divided into seven sections, structured as follows: Section 2 systematically outlines the three core technological routes for breathable electronics, conducting an in-depth analysis centered on the preparation of stretchable substrates, the construction of multi-scale breathability, and the conductive modification of devices. Section 3 discusses the core discrete components of breathable electronic systems, divided into two major sections: breathable interconnecting electrode wires and breathable energy devices, clearly identifying the advantages and limitations of different functional components in terms of conductivity, breathability, and cycling stability. Section 4 comprehensively summarizes the practical application progress of breathable electronics across various cutting-edge fields, sequentially introducing four major application areas: pressure/strain mechanical sensing, multi-channel electrocardiographic (ECG), electromyographic (EMG), electroencephalographic (EEG), and electro-oculographic (EOG) electrophysiological monitoring, implantable biochemical detection for the epidermis and wound surfaces, and multifunctional bionic electronic skin. Through case studies of typical devices, it analyzes the benefits of breathable structures for the long-term, high-fidelity acquisition of physiological signals. Section 5 discusses integration strategies for breathable electronics at the system level, focusing on a comparative analysis of the integration logic behind the two mainstream device architectures: fabric-integrated and patch-type. It also analyzes key technologies, such as signal conditioning circuits, wireless transmission, power supply, and packaging interfaces, and presents existing optimization approaches to address integration challenges, such as heterogeneous hardware-software interconnection, large-area fabrication, and protection of breathable channels. Section 6 critically examines the long-term failure mechanisms of breathable electronics in real-world scenarios, including sweat corrosion, mechanical wear, washing damage, and environmental aging, while highlighting state-of-the-art material, structural, and algorithmic strategies for reliability enhancement. Finally, Section 7 systematically reviews the core technical bottlenecks of breathable electronics and, informed by interdisciplinary trends, highlights future development directions. This chapter offers valuable R&D insights to bridge the gap toward the clinical and industrial application of this technology.

## 2. Design Strategies for Breathable Electronics

### 2.1. Fabrication of Stretchable Substrates

As the physical foundation for physiological integration, stretchable substrates must achieve a delicate balance between mechanical compliance, interfacial adhesion, and breathability to match human soft tissues. Currently, the fabrication strategies for stretchable substrates primarily diverge into two major material-based routes: intrinsically stretchable materials (encompassing high-performance elastomers and soft hydrogels) and structure-based mechanical designs (leveraging geometric configurations and topological engineering). This section systematically outlines these two core approaches, detailing how material topological control and macroscopic/microscopic structural innovations jointly resolve the long-standing conflict between mechanical stretchability and wearability.

#### 2.1.1. Intrinsically Stretchable Materials

Intrinsically stretchable substrates form the physical basis for the physiologically conformal integration of flexible electronic devices. Currently, intrinsically stretchable substrates achieve tissue-level compliance at the molecular level, primarily through two material systems: elastomers and hydrogels [42].

**Figure 2:**
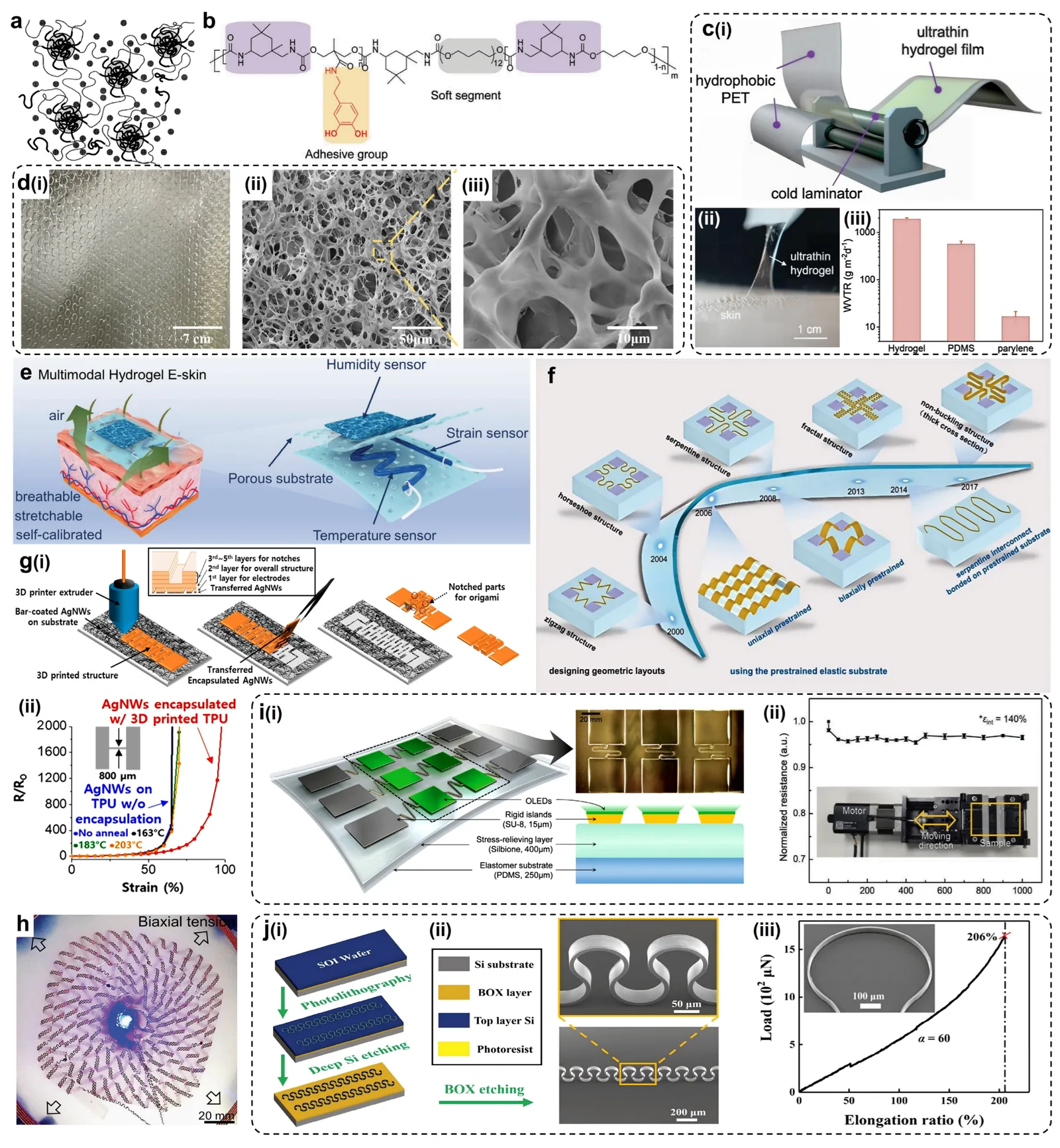
Strategies for Constructing Stretchable Substrates. (**a**) Schematic illustration of SEBS-dispersed micelles. Adapted from Ref. [43]. Copyright 1996, American Chemical Society. (**b**) Molecular structure of dopamine side chains. Adapted from Ref. [44]. Copyright 2021, WILEY. (**c**) 10 μm ultra-thin hydrogel film: (**i**) Schematic of the cold-pressing method, (**ii**) Demonstration of the hydrogel conforming to human skin, (**iii**) Comparative WVTR test conducted under 55% relative humidity. Adapted from Ref. [6]. Copyright 2023, WILEY. (**d**) Photomicrographs and scanning electron microscope (SEM) images of the origami-style ionic hydrogel. Adapted from Ref. [47]. Copyright 2022, Springer Nature. (**e**) Structure, properties, and structural components of the biomimetic multimodal hydrogel-based electronic skin. Adapted from Ref. [18]. Copyright 2024, WILEY. (**f**) Structural evolution of stretchable electronics. Adapted from Ref. [51]. Copyright 2023, WILEY. (**g**) Concealed origami configuration based on a 3D-printed setup: (**i**) fabrication process, (**ii**) strain of the system. Adapted from Ref. [21]. Copyright 2019, American Chemical Society. (**h**) Schematic illustration of a three-level 3D helical structure functioning as an electromagnetic coil to supply power to LEDs. Adapted from Ref. [52]. Copyright 2023, WILEY. (**i**) A double-layer cushioning substrate based on low-modulus Silbione: (**i**) Schematic of the hybrid stretchable platform and microscopic images of the SU-8-based rigid islands and serpentine interconnect structures fabricated on top of the Silbione layer, (**ii**) Dynamic stretch-release cycle testing of OLEDs. Adapted from Ref. [22]. Copyright 2020, WILEY. (**j**) Self-supporting serpentine silicon strips: (**i**) Schematic of the fabrication process for FS-Si strips, (**ii**) SEM image, (**iii**) Maximum elongation can reach 200%. Adapted from Ref. [23]. Copyright 2024, WILEY.

Elastomers, including thermosetting elastomers (TSE) and thermoplastic elastomers (TPE), are the mainstream materials for stretchable electronic devices, due to their excellent chemical stability and processability. Development strategies for these materials primarily focus on the molecular topological control of mechanical properties and the functionalization of interfaces. With regard to the topological control of mechanical properties, researchers tend to precisely match the low modulus requirements of human soft tissues by regulating the composition of polymer molecular segments. Steube et al. utilized anionic polymerization to prepare gradient block copolymers, achieving continuous adjustment of the TPE substrate modulus within the range of 20 kPa–12 MPa [8]. Laurer et al. modulated the microphase-separated morphology of Styrene-ethylene-butylene-styrene (SEBS) through mineral oil swelling (Figure 2a), producing an ultra-soft gel substrate with a modulus as low as 20 kPa [43]. Regarding the balance between interfacial adhesion and environmental adaptability, to address the challenge of electrode detachment during dynamic movement and in humid environments, Xu et al. employed dopamine side-chain grafting strategy. Driven by enhanced microphase-separated microstructure and moderate physical cross-linking from catechol-related interactions, the elastomer obtained an ultrahigh fracture strain of 5100%, accompanied by room-temperature self-healing capability and high wet adhesion (16 kPa) (Figure 2b) [44]. Nan et al., focusing on mucosal applications, established a design threshold requiring wet adhesion of 20 kPa [45]. Furthermore, to address skin irritation caused by the lack of breathability in elastomers, Dong et al. utilized electrospinning to construct a SEBS nanofiber network, combined with perfluorinated hydrophobic modification, this achieved good breathability and self-cleaning properties [46]. It is thus evident that elastomer development has increasingly shifted from the independent optimization of stretchability toward the coordinated regulation of modulus, adhesion, durability, and physiological compatibility.

Hydrogels are attractive for biointerface integration because their high water content, ionic conductivity, and low Young’s modulus resemble the properties of biological tissues. However, their practical use may be limited by insufficient mechanical strength, dehydration, and environmental instability. Reducing the film thickness and introducing supporting or interfacial layers can improve conformability while maintaining adequate physiological compatibility. Cheng et al. innovatively prepared an ultra-thin 10 μm hydrogel film using a cold lamination method (Figure 2c(i)), ensuring both a perfect fit with skin texture (Figure 2c(ii)) and excellent breathability (Figure 2c(iii)) [6]. In addition, geometric and microstructural engineering can enhance the mechanical robustness and sensing functionality of hydrogel substrates. Xu et al. combined Kirigami structures with ionic hydrogels to achieve dual tactile and strain sensing whilst maintaining the comfort of the fabric (Figure 2d) [47]. Wang et al., through hydrogel microstructural engineering (Figure 2e), developed a self-calibrating multimodal skin that effectively resolved the issue of crosstalk between multi-parameter signals, such as strain, temperature, and humidity, achieving a strain detection limit as low as 0.03% [18]. By designing micro/nanoscale geometries, researchers have addressed hydrogels’ intrinsic drawbacks in mechanical strength and anti-interference, achieving advanced sensing functions. Accordingly, hydrogel material development centers on leveraging micro/nanostructures to overcome inherent mechanical limitations and optimizing interfacial layers for physiological permeability, to fabricate breathable, stretchable substrates.

Overall, intrinsically stretchable materials are most suitable when continuous surface coverage, conformal contact, and scalable processing are prioritized. Elastomers generally provide better environmental stability and manufacturing compatibility, whereas hydrogels offer softer and more tissue-like interfaces but require further improvement in mechanical durability and long-term stability.

#### 2.1.2. Structure-Based Design

The key to realizing stretchable electronic systems lies in constructing a mechanical structural system capable of withstanding mechanical deformation while maintaining high-efficiency electrical performance. As epidermal electronics have evolved from simple physiological signal monitoring to high-density multifunctional system integration, the scope of structural design has expanded from simple tensile-resistant interconnections to system-level architectural synergy. The central principle is to redistribute external deformation through geometry, thereby reducing the strain transferred to rigid functional components and improving device reliability [48,49]. Currently, structured stretchable substrates are primarily constructed using the following geometric configurations: Researchers utilize structures such as serpentine, helical, lattice, pleated, origami, and three-dimensional (3D) hierarchical architectures (Figure 2f) to effectively decouple macroscopic external loads and convert them into localized structural deformations. These configurations convert tensile deformation into localized bending, buckling, rotation, or unfolding, thereby reducing stress-induced damage in active materials such as silicon thin films, nanowires, and perovskites. Based on this design paradigm, the specific implementation paths for structured designs are primarily divided into the following four evolutionary directions.

First, buckling and serpentine interconnections provide an in-plane geometric strategy for strain accommodation. Wavy or serpentine conductors induce out-of-plane buckling during stretching, thereby converting tensile strain into bending strain. Pre-straining and controlled over-stretching can further extend the elastic operating range of metallic interconnects [50,51]. Because of their relatively simple planar configuration and processing compatibility, these structures are particularly suitable for mainly uniaxial or moderate biaxial deformation. Thickness-dependent crease patterns provide another route for improving in-plane extensibility [49].

Second are spatial-dimension-based origami/paper-cutting structures and 3D hierarchical architectures. As applications have evolved from uniaxial to multiaxial deformation, simple planar structures have become inadequate for handling complex deformations. Researchers have developed origami and paper-cutting structures by expanding into the spatial dimension. Jo et al. used 3D printing technology to design a concealed origami configuration that releases strain through a controlled unfolding path (Figure 2g(i)), achieving up to 400% system stretch (Figure 2g(ii)) while maintaining an extremely high active coverage ratio [21]. Furthermore, the biomimetic 3D hierarchical (Figure 2h) design proposed by Zheng et al. successfully overcomes the stress concentration issues of conventional helical structures under multiaxial stretching by transforming two-dimensional (2D) serpentine lines into multilevel helices [52]. These designs are advantageous for complex deformation but generally require more sophisticated geometric modeling, fabrication, and quality control.

Third is the island-bridge layout scheme based on stiffness gradients and stress diversion. Electronic units such as organic light-emitting diodes (OLEDs), memory elements, and microprocessors are placed on relatively rigid islands and connected by stretchable bridges. Buffer layers and stiffness-gradient designs reduce the strain transferred to the functional islands, thereby improving the mechanical stability of integrated devices. Taehyun Kim significantly alleviated interfacial stress in pixel islands by constructing an ultra-low-modulus Silbione double-layer buffer substrate (Figure 2i(i)), resulting in minimal performance degradation of OLEDs under tension (Figure 2i(ii)) [22]. The theoretical core of this strategy lies in suppressing the “island effect” through stiffness gradient optimization, so it is particularly suitable for high-density systems containing rigid components, although its performance depends on island dimensions, bridge spacing, encapsulation thickness, and stiffness distribution [53,54].

Finally, there is the strategy based on microporous and self-supporting network structures. This approach balances mechanical stretchability with breathability and heat dissipation during wear. By introducing microporous arrays into the substrate or employing self-supporting fabrication techniques, this approach not only reduces the structure’s equivalent stiffness, but also addresses the issues of sweat accumulation and skin wetting caused by long-term wear of epidermal electronics. Li et al. fabricated circular microporous PDMS structures using a template method, which significantly improved interfacial breathability while achieving a substantial increase in the strain ratio (from 5% to 45%) [55]. On the other hand, by removing substrate constraints, the self-supporting serpentine silicon strips fabricated by Su et al. enabled micro- and nanostructures to exhibit a maximum strain exceeding 200% during out-of-plane movement (Figure 2j), demonstrating outstanding performance in high-performance flexible optoelectronic detectors [23]. These structural solutions fully reflect the profound evolution of mechanical design toward multi-physics compatibility and serve as the foundation for achieving long-term, stable, and reliable operation of “skin-like” electronic devices.

The above evolutionary pathways demonstrate that the mechanical design paradigm of flexible electronics has shifted from passive stress relief toward active structural control [56,57]. Planar serpentine and buckling structures provide basic strain accommodation; origami, kirigami, and hierarchical 3D architectures address large and multiaxial deformation; island-bridge layouts improve the reliability of systems containing rigid functional units; and microporous or self-supporting structures combine low stiffness with improved physiological compatibility. Future structural designs are expected to further integrate these functions through additive manufacturing and advanced multilayer fabrication, thereby improving system integration, service life, and human–machine interaction [17].

#### 2.1.3. Comparative Evaluation and Application Scenarios

The stretchable-substrate strategies in Table 1 differ in deformation capability, reliability, fabrication complexity, and scalability. Elastomers provide processability, environmental stability, and strong adhesion, whereas hydrogels offer lower modulus and tissue-like interfaces but remain limited by dehydration and mechanical durability. Serpentine structures provide a simple solution for mainly uniaxial deformation, while origami, kirigami, and hierarchical 3D architectures enable larger and multiaxial stretching at the cost of fabrication complexity. Island-bridge designs prioritize the protection of rigid functional components in integrated systems. As shown in Table 1, maximum elongation does not determine overall performance: dopamine-modified elastomers achieve high extensibility, hydrogels provide excellent compliance, and structural designs offer application-specific advantages in strain accommodation or device integration. Across these approaches, maximum strain alone is insufficient for comparison; cyclic stability, environmental resistance, interfacial integrity, and manufacturing scalability should also be considered.

In practical applications, material-based substrates are preferred when conformability, continuous coverage, and scalable processing are required, whereas geometric architectures are more appropriate for high-strain or multiaxial deformation. The final selection should therefore consider working strain, cyclic durability, interfacial stability, active-area coverage, device integration, and manufacturing scalability together rather than relying on the maximum reported elongation alone.

**Table 1:** Overview of stretchability, advantages, and disadvantages of various stretchable strategies.

Design Dimension	Construction Strategy	Stretchability	Advantages	Disadvantages	Refs.
Elastomers	Dopamine-modified elastomers	5100%	Strong adhesion, room-temp self-healing	Poor thermal stability	[44]
Hydrogels	Ultrathin laminated hydrogels	High compliance	Universal interface, breathable	Low mechanical strength, prone to drying	[6]
Serpentine/Buckling	Pre-strained serpentine interconnects	185%	Significantly broadens elastic stretch range	Planar configuration, poor multi-axial capability	[50]
3D/Origami	Hierarchical 3D helical structures	>200%	Multi-axial and isotropic stretching	Complex geometric modeling, time-consuming	[52]
3D/Origami	3D-printed hidden origami	400%	System-level high areal coverage	Micro-scale precision and mass production	[21]
Island-Bridge	Bilayer buffering stiffness gradient	350%	Effective pixel island protection, brightness stability	Low substrate strength, mechanical damage	[22]

Note on Table 1: Fracture strain (stretchability, ε_b_) is defined as: $\varepsilon_{b}=\frac{L_{b}-L_{0}}{L_{0}}\times 100\%$ (where L_0_ is the initial gauge length and L_b_ is the length at break).

In dopamine-modified polyurethane elastomers [45], the ultra-high 5100% stretchability is fundamentally enabled by the highly flexible polytetramethylene glycol soft segments and dynamic hydrogen-bonding/π-π network that dissipates mechanical energy under elongation. The pendant dopamine moieties primarily serve to provide robust wet/dry tissue adhesion and room-temperature self-healing, rather than acting as the sole driver for stretchability.

### 2.2. Breathable Design

Traditional dense substrates can hinder heat and moisture exchange at the skin interface, thereby reducing long-term wearing comfort and device reliability. To address this limitation, breathable electronic substrates have evolved from simple material modification toward multiscale structural engineering. Current strategies can be broadly classified into three categories: intrinsically breathable textile and polymeric substrates, pore-structured materials, and electrospun fibrous membranes. These approaches improve physiological compatibility through different mechanisms, including wettability regulation, interconnected gas-transport channels, and multiscale porous networks.

#### 2.2.1. Breathable Textile Substrate

Traditional flexible electronic devices often use dense elastomers (such as PDMS and PU films) as substrates. These materials have extremely low WVP, causing sweat to accumulate at the interface and leading to skin inflammation and electrical signal drift [58,59,60]. The development of electronic materials with “intrinsic breathability”, which refers to material systems that possess micron-scale molecular permeation channels within their structure while maintaining high electrical conductivity, has become the key approach to addressing wearability issues [58,61]. The strategies for developing such materials can be broadly categorized into the following three aspects.

**Figure 3:**
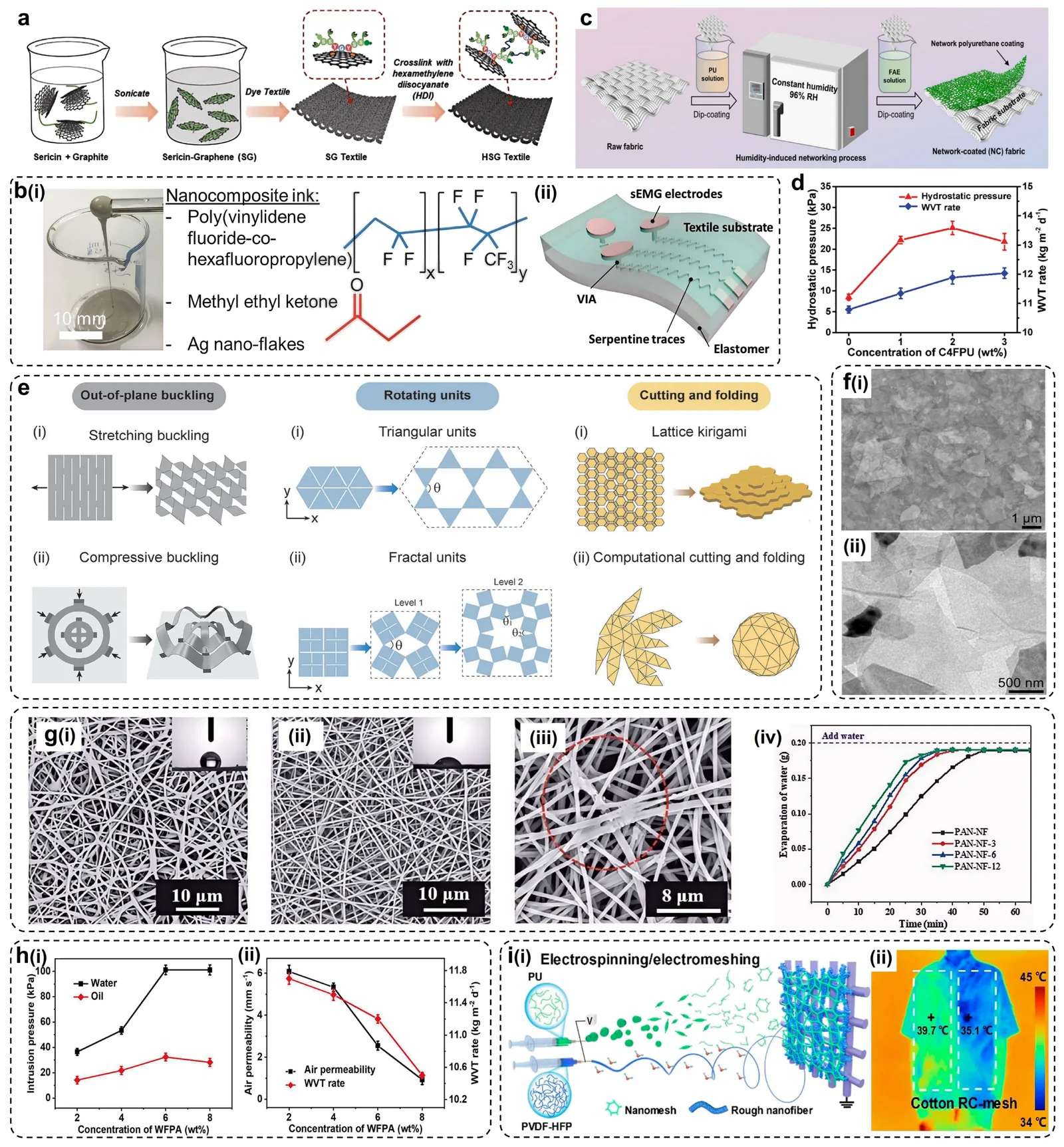
Strategies for Achieving Breathability. (**a**) Preparation process for hydrophilic conductive fabrics developed based on a filament protein-assisted graphene aqueous dispersion system. Adapted from Ref. [61]. Copyright 2022, WILEY. (**b**) Silver-particle-fluoropolymer composite ink: (**i**) composition and chemical structure, (**ii**) schematic diagram of the prepared bilayer electronic patch. Adapted from Ref. [59]. Copyright 2018, WILEY. (**c**) Schematic diagram of the construction of a PU porous network via the humidity-induced web-forming method. Adapted from Ref. [24]. Copyright 2026, Springer Nature. (**d**) PU fiber membranes containing short-chain perfluorobutyl groups achieve a water pressure resistance of 102.8 kPa and a high air permeability of 12.9 kg/(m^2^·d). Adapted from Ref. [58]. Copyright 2018, American Chemical Society. (**e**) Overview of engineered paper-cutting framework technology. Adapted from Ref. [25]. Copyright 2024, WILEY. (**f**) VDWTFs assembled from interlaced 2D nanosheets: (**i**) SEM image, (**ii**) TEM image. Adapted from Ref. [26]. Copyright 2022, American Association for the Advancement of Science. (**g**) SEM images: (**i**) TPU, (**ii**) PAN, (**iii**) PAN & dopamine (PDA), (**iv**) unidirectional water transport rate is three times higher than that of conventional films. Adapted from Ref. [76]. Available under a CC-BY 3.0 license. (**h**) Performance of eco-friendly membranes developed using ethanol-solvent spinning technology: (**i**) capable of withstanding a high water pressure of 101.2 kPa, (**ii**) capable of maintaining a high moisture permeability rate of 11.2 kg m^−2^·d^−1^. Adapted from Ref. [75]. Copyright 2021, American Chemical Society. (**i**) RC-mesh membrane: (**i**) schematic diagram of synthesis, (**ii**) demonstration of infrared radiation cooling function. Adapted from Ref. [27]. Copyright 2023, American Chemical Society.

First, the wettability of the material determines the efficiency of sweat conduction. Liang et al. developed a hydrophilic conductive fabric using a silk protein-assisted aqueous graphene dispersion system (Figure 3a), with an average graphene layer thickness of approximately 4 nm [61]. While maintaining a stable conductive pathway, this material utilizes the hydrophilic groups of silk protein to facilitate sweat diffusion, ensuring the stability of the device’s interfacial impedance under physiological conditions. Similarly, La et al. used a composite ink of silver particles and fluoropolymer to impregnate a fabric substrate (Figure 3b), resulting in a bilayer electronic patch that exhibited excellent water absorption properties, with a water absorption rate of up to 18.5 g/g for the textile substrate [59]. In EMG monitoring, this system performed comparably to commercial gel electrodes, and its breathability ensured that signals remained free of baseline drift during long-term testing.

Second, endowing materials with intrinsic porosity through molecular-level structural regulation is another key approach to achieving a balance between high breathability and high performance. Zhou et al. used a humidity-induced web-forming method (Figure 3c) to construct a PU porous network with a porosity as high as 62.1%, achieving a water vapor permeability rate (WVPR) of 2764.7 g/(m^2^·d), effectively balancing breathability with waterproofing and windproofing requirements [24]. Under conditions of higher pressure and high humidity, the PU fiber membrane containing short-chain perfluorobutyl groups developed by Zhao et al. demonstrated a water pressure resistance of 102.8 kPa and a high air permeability of 12.9 kg/(m^2^·d) (Figure 3d) [58]. The main advantage of this approach is that permeability is incorporated into the material structure rather than relying solely on macroscopic openings.

Finally, breathable conductive materials must also retain their electrical and mechanical performance during deformation. The electronic fabric prepared by Li et al. [62] via pneumatic-assisted screen printing achieved a sheet resistance as low as 82.16 Ω/sq, while its breathability decreased by only 27.8% compared to the original fabric, demonstrating excellent process compatibility. Furthermore, this strain sensor achieved a sensitivity factor of 36 over a 0–100% stretch range and remained stable after 9000 stretch cycles, demonstrating that intrinsically breathable materials possess excellent electromechanical coupling durability while delivering high-performance sensing capabilities. Fabric antennas and multimaterial fibers can also support contactless physiological monitoring while preserving the original textile structure [63]. Thus, intrinsically breathable textiles are particularly promising for large-area and conformal devices, although the long-term stability of conductive components under repeated stretching, washing, and moisture exposure remains to be established.

Overall, textile and polymeric substrates provide a relatively continuous device surface and good process compatibility. Their main challenge is to maintain a stable balance among electrical conductivity, mechanical durability, and moisture transport during long-term use, especially when porous matrices are combined with high-performance electronic materials.

#### 2.2.2. Breathability Design Based on Pore Structure

By introducing a controlled pore structure into a substrate or conductive network, an originally dense electronic material can be transformed into a functional body with highly efficient gas exchange channels. Based on the scale and mechanism of pore formation, existing breathability design strategies can be broadly categorized into two types: macrostructural enhancement and microscopic pore-based physical permeation [19,64,65].

Macroscopic porosity is typically achieved by pre-forming spatial channels on the device surface or substrate through specific patterning processes, such as paper-cutting, fractal cutting, or 3D assembly. The paper-cutting technique is a typical representative of this strategy, by introducing periodic cutting patterns, it transforms traditional 2D thin films into flexible metamaterials with excellent gas permeability. The geometric rules of honeycomb origami provide a basis for constructing 3D topologies from planar sheets [66], while fractal cutting enables multiscale pore arrangements and improves deformation adaptability (Figure 3e) [25]. At the macroscopic level, pore formation simultaneously provides ventilation pathways and releases mechanical strain, making it useful for devices that must accommodate large deformation. Furthermore, mechanically guided 3D assembly techniques have further expanded the dimensions of breathable design: Bo et al. summarized a buckling-guided 3D assembly strategy based on pre-stretched substrates that induce the out-of-plane buckling of 2D precursors, enabling the conversion of planar electronic structures into 3D architectures and providing a methodological foundation for the design of stretchable breathable electronics [56]. At the macroscopic level, pore design leverages the coupling of topological deformation and geometric channels to achieve efficient synergy between the device’s tensile toughness and gas permeability.

Unlike the morphological evolution of macroscopic paper-cutting structures, microscopic pore strategies aim to construct nanoscale gas transport channels through structural assembly at the material level, thereby achieving “intrinsic breathability” without compromising material integrity. Yan et al. proposed a strategy for preparing van der Waals films (VDWTFs), utilizing the self-assembly of 2D nanosheets after liquid-phase exfoliation to construct a nanoscale pore system with an average pore diameter of approximately 10 nm (Figure 3f) [26]. This system exhibits a WVTR as high as 34 mg cm^−2^ h^−1^, balancing ultra-high tensile strength with excellent gas permeability. Inkjet-printed 2D heterojunctions similarly use the interconnection of nanosheets to generate percolating nanopores, providing a potentially scalable route for conformal electronic devices [67]. Compared with macroscopic cut-out structures, nanoporous systems can preserve a larger active area, but their pore stability, contamination resistance, and uniformity during long-term deformation require further validation.

Macroscopic cut-out designs provide deformation release and motion adaptation capabilities through spatial geometric transformations. However, their complex cutting processes limit production efficiency and pattern accuracy in large-area electronic devices [68,69]. The micro-nanopore strategy focuses on building the material’s intrinsic permeability, but structural collapse of the nanoporous structure under long-term dynamic deformation and its filtration performance regarding sweat impurities remain key challenges limiting its long-term clinical application [70]. In the future, a composite design incorporating hierarchical, multi-scale pore structures, combined with dynamic fatigue life prediction models, will be a major research trend for overcoming these bottlenecks and developing long-lasting, high-performance breathable electronic devices [20,71].

#### 2.2.3. Breathable Spun Bond Film

As a porous nonwoven fabric composed of randomly stacked nanoscale fibers, breathable electrospun membranes have become an ideal substrate for constructing unobtrusive interactive interfaces in the field of flexible electronics due to their extremely high porosity and excellent structural tunability.

Electrospun fibrous membranes consist of interconnected micro- and nanoscale fibers, forming a 3D network with high porosity and a large specific surface area. Their breathability mainly originates from the open pathways between fibers, while their fiber diameter, surface chemistry, and layer arrangement can be adjusted to control transport and interfacial functions [72,73]. Building on this, Gong et al. [74] utilized a humidity-induced biomimetic hierarchical structure to form nanoscale protrusions on the fiber surface. While maintaining a high WVTR of 11.2 kg/(m^2^·d), they achieved a superhydrophobic contact angle of 154.6°, significantly enhancing the membrane’s self-cleaning capability and preventing electronic failures caused by sweat accumulation. Compared to dense polymer films, the open-cell structure of spun-fiber membranes allows the material to engage in direct microfluidic exchange with human skin, thereby effectively alleviating the occlusion effect that occurs at the skin interface with traditional electronic substrates [75].

In real-world wearing scenarios, breathability alone is insufficient to address sweat accumulation during high-intensity exercise. Therefore, developing breathable spun-fiber membranes with a wettability gradient is key to enhancing wearing comfort. Zhang et al. [76] ingeniously combined a hydrophobic thermoplastic polyurethane (TPU) inner layer with a hydrophilic polyacrylonitrile (PAN) outer layer by fabricating a Janus-structured membrane (Figure 3g(i)–(iii)). By leveraging the difference in wettability between the two sides, they achieved unidirectional sweat wicking (Figure 3g(iv)), resulting in a moisture permeability rate as high as 9065 g/(m^2^·d) and an air permeability of 100 mm s^−1^ (5 times higher than a commercial membrane). Building on this, Zheng et al. [77] further utilized ionic liquids to functionalize the spun fibers, developing an ionic-liquid-functionalized spun membrane. While enhancing unidirectional moisture transport efficiency, this membrane exhibited an antibacterial rate of up to 99% against Staphylococcus aureus, effectively addressing the challenge of skin inflammation commonly associated with long-term monitoring and achieving a three-in-one design that integrates breathability, moisture transport, and antibacterial properties.

Electrospun membranes can also integrate additional functions, including antibacterial protection, water resistance, environmental durability, and thermal management. Ionic-liquid functionalization, for instance, combines directional moisture transport with antibacterial activity [75]. Yang et al. prepared a fabric by compositing highly water-absorbent fibers, achieving a moisture permeability rate of 12,638.5 g/m^2^·d, approximately three times that of traditional protective fabrics, and nearly doubling the moisture absorption rate, thereby enabling the seamless integration of high-performance medical protective clothing with real-time physiological monitoring [78]. Gong et al. developed a multi-level nanomesh fabric (Figure 3i(i)) that, while providing a high water pressure barrier of 95.6 kPa, integrates infrared radiation cooling functionality (Figure 3i(ii)), reducing the human body’s surface temperature by up to 7.9 °C, thereby establishing a new paradigm for smart thermal management electronics [27]. These capabilities make electrospun membranes particularly suitable for skin-contact devices that require simultaneous ventilation, moisture management, and functional integration.

In summary, breathable spun-bonded membranes achieve a balance of high porosity, high specific surface area, and excellent mechanical flexibility at the micrometer scale through the physical reorganization of nanofibers, endowing the electronic substrate with active sweat management and passive gas exchange capabilities [79]. However, the open fiber network may experience fatigue during repeated stretching, and conductive or functional components may detach from the fiber surface during deformation or washing [27,73,80]. Future development should therefore focus on improving mesh durability, interfacial bonding, environmental resistance, and the uniform integration of functional components [81,82].

#### 2.2.4. Comparative Evaluation and Application Scenarios

The breathability strategies discussed above differ in transport mechanism, device compatibility, and manufacturing potential. The data in Table 2 illustrate that the reported breathability values should be interpreted together with the transport mechanism and functional advantages of each design. Textile and polymeric substrates provide a balance between conductivity, flexibility, and breathability, making them appropriate for wearable sensors and fabric-integrated electronics. Nanoporous 2D films offer high permeability while preserving material continuity and tensile performance, which is beneficial for conformal electronic interfaces but may complicate uniform large-area fabrication. Janus fibrous membranes provide directional moisture transport rather than simple passive permeability, giving them a practical advantage in skin-contact applications. Multilevel nanofiber networks additionally integrate thermal management, whereas green solvent-based membranes and sea-island fibers emphasize environmental compatibility and scale-up potential.

**Table 2:** Overview of the Air Permeability, Advantages and Disadvantages of Various Air Permeability Design Methods.

Design Dimension	Construction Strategy/Method	Breathability Metric	Advantages	Disadvantages	Refs.
Breathable Fabric Substrate	Pneumatically assisted screen printing (Graphene)	38.5 L/(m^2^·s)	Good balance of conductivity and breathability	Lack of sweat immersion stability test	[62]
Breathable Fabric Substrate	Humidity-induced porous network coating	2764.7 g/(m^2^·d)	Combines windproof, waterproof, and breathable properties	Durability in washing/abrasion needs validation	[24]
Breathable Fabric Substrate	Spiral antenna/multi-material fiber integration	Maintains original fabric breathability	Non-contact monitoring, no skin contact needed	Cannot monitor detailed parameters like tidal volume	[63]
Porous Structure	Nanoporous 2D material thin films	34 mg cm^−2^ h^−1^	Ultra-high stretchability and breathability	Challenges in large-scale uniformity	[26]
Porous Structure	Nanoporous metallized polyethylene	12 mg cm^−2^ h^−1^	Infrared reflection combined with breathability	Reduced material flexibility	[83]
Breathable Fibrous Membrane	Janus dual-layer nanofiber membrane	100 mm/s	Efficient directional moisture transport	Lack of washability/durability testing	[76]
Breathable Fibrous Membrane	Fluorine-free eco-friendly multifunctional coating	10.3 kg/(m^2^·d)	Eco-friendly, antibacterial, UV-resistant	Coating interface durability needs improvement	[80]
Breathable Fibrous Membrane	Multi-level nanofiber network	36.7 mm/s	Integrates radiative cooling with waterproof breathability	Nanostructures prone to collapse after washing	[27]
Breathable Fibrous Membrane	Green solvent-based fibrous membrane	2.6 mm/s	Low-toxicity production, water/dust repellent	No washability testing conducted	[75]
Breathable Fibrous Membrane	Sea-island melt-spun ultrafine fibers	284 mm/s	Suitable for industrial-scale production	Aerogel prone to shedding after washing	[73]

The numerical values in Table 2 should not be used to establish a direct ranking because the reported metrics differ in definition and may depend on humidity, pressure, thickness, sample geometry, and testing conditions. In practical selection, continuous textile or polymer platforms are preferable when electrical integration and large-area coverage are prioritized; macroporous structures are suitable for large deformation and direct ventilation; nanoporous materials are advantageous when material continuity must be preserved; and electrospun membranes are most attractive when directional moisture transport or multifunctional skin-interface management is required. Thus, breathable-substrate selection should consider transport mechanism, active-area coverage, mechanical durability, functional integration, environmental resistance, and manufacturing scalability rather than permeability values alone.

### 2.3. Strategies for Forming Conductive Pathways

Transforming insulating, breathable substrates into functional electronic devices requires establishing continuous and stable conductive pathways while preserving porous gas-exchange channels. In this review, conductive-pathway construction refers to the material modification and fabrication strategies used to impart electrical conductivity to initially insulating breathable substrates or to establish continuous conductive networks within them. These strategies should maintain electrical continuity during mechanical deformation without blocking the gas- and moisture-transport channels required for breathability. This section categorizes these conductive-pathway construction strategies into three main paradigms: intrinsic conductive materials (utilizing conjugated polymers, metal nano-percolation networks, and 2D MXene nanosheets for molecular-level conductivity), and physical fabrication methods (employing physical vapor deposition (PVD), transfer printing, and template-assisted techniques to construct robust interfacial conductive layers), and chemical construction methods (employing in situ polymerization, chemical plating, and interfacial chemical bonding to form robust conductive networks).

#### 2.3.1. Intrinsic Conductive Materials

The use of intrinsically conductive materials provides a direct route to imparting electrical functionality to flexible substrates. The main material systems include intrinsically conductive polymers, metal nanowire networks, and 2D materials. Their suitability depends on the balance among conductivity, stretchability, environmental stability, and interfacial adhesion.

**Figure 4:**
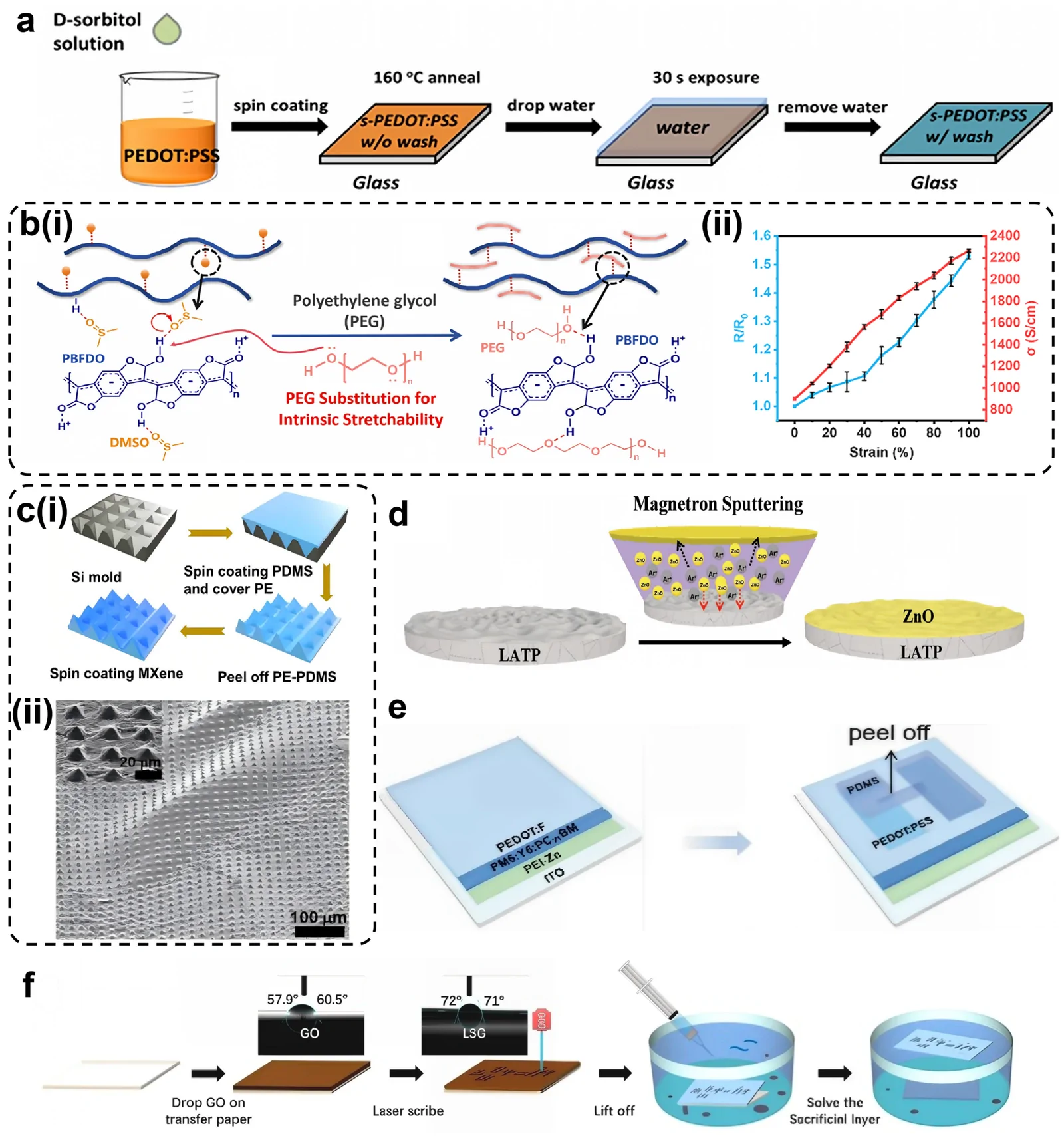
Strategies for enhancing conductivity. (**a**) Process flow for introducing the biocompatible small molecule D-sorbitol to improve the elongation at break of PEDOT:PSS. Adapted from Ref. [85]. Copyright 2019, American Chemical Society. (**b**) Enhancing conductivity through secondary doping and molecular plasticization: (**i**) PEG plasticization strategy, (**ii**) a conductivity of 2265 S/cm was achieved at 100% strain. Adapted from Ref. [28]. Copyright 2026, WILEY. (**c**) Conductive modification based on MXene materials: (**i**) Preparation process for MXene-PDMS-PE films (MPP), (**ii**) SEM image of a substrate coated with MXene nanosheets. Adapted from Ref. [29]. Copyright 2022, American Association for the Advancement of Science. (**d**) Interface evolution between the lithium metal anode and LATP. Adapted from Ref. [92]. Copyright 2019, WILEY. (**e**) Schematic illustration of transferring the PEDOT:PSS film onto the PEDOT:F film. Adapted from Ref. [30]. Copyright 2022, WILEY. (**f**) The process of fabricating graphene with the Schrodinger equation on transfer paper and the contact angles of GO and LSG. Adapted from Ref. [31]. Copyright 2018, American Association for the Advancement of Science.

Organic semiconductors, particularly conjugated polymers, provide a flexible material platform in which charge transport can be regulated through molecular design, intermolecular packing, and doping [84]. Unlike the band-like transport commonly associated with ordered inorganic semiconductors, charge transport in many organic materials is strongly affected by localized states and structural disorder. Their solution processability and low-temperature compatibility are advantageous for thin and fibrous conductive pathways, although conductivity, environmental stability, and mechanical durability remain important limitations. Conductive polymers such as PEDOT:PSS can therefore be mechanically modified through secondary doping, molecular plasticization, and hydrogen-bond network formation. The incorporation of biocompatible plasticizers can increase the elongation of PEDOT:PSS from approximately 8% to more than 60% while maintaining conductivity above 1000 S/cm, enabling the material to penetrate or coat textile substrates (Figure 4a) [85]. Similarly, Kim et al. utilized a hydrogen-bonded bridging network induced by the protonic ionic liquid p-MIM:TCB to prepare flexible films with a crack initiation strain exceeding 50%, successfully endowing the devices with excellent dynamic mechanical stability [86]. Furthermore, regarding the electrification of n-type polymers, the polyethylene glycol (PEG) plasticization strategy proposed by Zhao et al. (Figure 4b(i)), which induces interlayer intercalation via ether-oxygen bonds (Figure 4b(ii)), increased the elongation at break of PBFDO by a factor of 8 and achieved an excellent conductivity of 2265 S/cm at 100% strain, effectively filling the gap in complementary circuits for breathable electronic applications [28]. Although such methods exhibit excellent biocompatibility, their conductive stability in long-term humid and hot environments remains limited by the loss of dopants or environmental degradation [87].

Metal nanowire permeation networks provide another highly efficient pathway for breathable electronics by constructing a randomly distributed conductive grid on a breathable substrate. This method primarily relies on the high aspect ratio of metal nanowires (AgNWs and CuNWs), enabling conductive permeation even at extremely low loading levels while preserving the physical porosity of the substrate. A systematic review by Joo et al. indicates that using Joule heating or capillary-assisted cold welding techniques can significantly reduce nanowire junction resistance, enabling AgNW networks to maintain a conductivity of approximately 4692 S/cm even under 200% strain [88]. To balance electrical performance with optical transparency, Cama et al. [89] fabricated metal mesh electrodes using the crack-template method, achieving a low sheet resistance of 0.28 Ω/sq without the need for indium tin oxide (ITO). Furthermore, by adjusting process parameters, these electrodes could be transferred intact to a breathable PDMS substrate. While these methods excel in high-performance acoustic sensing and electrophysiological recording, their primary bottlenecks lie in the risk of metal component oxidation and stress concentration during dynamic cycling, typically requiring secondary optimization through core–shell structures or composite encapsulation [90].

Emerging 2D materials such as MXene, with their metal-level conductivity and hydrophilicity, demonstrate unique conductive advantages through microstructural integration. Gou et al. spin-coated Ti_3_C_2_Tx MXene onto an array of micro-pyramids on a PDMS substrate (Figure 4c), by leveraging the large interlayer spacing of 1.31 nm and the geometric deformation of the array to generate a two-stage amplification effect, they achieved an ultra-low detection limit of 0.1 Pa, providing a model for the construction of high-performance electronic skin [29]. However, the highly reactive surface of MXene makes it prone to oxidative degradation, achieving long-term stable chemical modification and patterning on large-area, breathable substrates remains a core challenge for future application and commercialization in this field [29,91].

Overall, intrinsic conductive materials integrate electrical functionality directly into the substrate or its surface. Conductive polymers are suitable when compliance and solution processing are prioritized, metal nanonetworks when low resistance and transparency are required, and 2D materials when high sensitivity and structure-enabled amplification are important. Their common challenge is maintaining stable interfaces and conductivity under environmental exposure and repeated mechanical cycling.

#### 2.3.2. Physical Construction Method

Physical construction methods integrate preformed conductive materials with flexible substrates through deposition, transfer, coating, or template-assisted processing. Compared with direct chemical synthesis, these methods provide more precise control over conductor placement, layer thickness, and interfacial morphology, making them useful for patterned electrodes and heterogeneous electronic systems.

PVD, particularly magnetron sputtering, can produce uniform functional layers and improve interfacial contact. For example, Hao et al. used magnetron sputtering to form a ZnO buffer layer on the surface of a lithium aluminum titanium phosphate (LATP) solid-state electrolyte (Figure 4d). Through an in situ reaction that induced the formation of an interfacial solid electrolyte interphase (SEI) layer, they successfully reduced the interfacial impedance from 80 kΩ to 353 Ω, effectively resolving the interfacial contact challenge between rigid substrates and electrodes [92]. In breathable electronic applications, sputtering not only enables the fabrication of transparent conductive oxides (such as SnO_2_) as electrodes [93], but also allows for the precise control of process parameters (such as substrate temperature and argon-to-oxygen ratio) to regulate the film’s crystallinity and work function [94]. However, its principal limitation is the brittleness of deposited films, which makes structural strain relief or additional interfacial modification necessary for highly deformable devices.

Transfer printing is a key process for the non-destructive integration of complex conductive structures onto breathable substrates. This method uses a sacrificial layer or a PDMS stamp to transfer pre-fabricated high-performance conductive films (such as Au, Pt, and AgNWs) from a master substrate to a target breathable substrate. Sacrificial-layer and water-assisted transfer methods have been used to place metal films and silver nanowire networks on flexible or curved surfaces. Surface treatment and buckling designs can improve adhesion and preserve conductivity under tensile deformation [95,96]. Furthermore, Wei et al. [30] proposed a lamination transfer process for a double-layer conductive polymer top electrode, in which a high-work-function PEDOT:F layer was stacked with a highly conductive PEDOT:PSS layer with the aid of PDMS (Figure 4e). This approach achieved an energy conversion efficiency of 12.46% in non-fullerene organic solar cells, demonstrating the advantages of transfer technology in the integration of complex multilayer electronic systems [31]. Transfer-based methods are particularly effective for heterogeneous integration, although their yield and reliability depend strongly on surface chemistry, thickness control, and the stability of the transferred interface.

Physical coating and mold-assisted methods have further expanded the scope of physical fabrication. Chen et al. innovatively coated liquid metal (LM) (Ga-based alloy) droplets with graphene flakes and utilized self-assembly techniques to construct a non-viscous, non-corrosive conductive soft-contact system [97]. This method not only preserves the high electrical conductivity of the LM, but also, through the graphene shell, enables the droplets to roll within microchannels, offering a new approach for mobile soft-contact electronic devices. In terms of electrode patterning, physical processes based on anodic aluminum oxide (AAO) films or PDMS transfer templates can achieve submicron-resolution patterned deposition, effectively addressing the issue of uncontrolled morphology of conductive materials in traditional wet-process fabrication [98].

In summary, physical methods are most valuable when precise conductor placement, multilayer integration, or interfacial control is required. Although physical construction methods have performed exceptionally well in laboratory-scale device prototyping, challenges related to large-area, roll-to-roll (R2R) processing of breathable substrates, as well as interface adhesion degradation under long-term dynamic stretching, still need to be addressed through the introduction of more advanced interface modification techniques (such as plasma pretreatment) [95,96].

#### 2.3.3. Chemical Synthesis Method

Chemical synthesis methods generate conductive networks directly on or within the substrate through laser-induced transformation, electrochemical deposition, electroless plating, or in situ polymerization. By combining material formation with patterning, these approaches reduce the need for a separate conductor-transfer step and enable substrate-specific device fabrication.

Laser-induced chemical transformation is the core technology for making breathable substrates conductive. Tour et al. were the first to propose using a CO_2_ laser to induce photothermal carbonization of polyimide (PI) films, directly generating laser-induced graphene (LIG) [99]. Building on this, Qiao et al. [31] further developed LIG lift-off and direct-writing techniques to precisely fabricate all-graphene patterns on flexible paper substrates. This technology, through precise control of the laser path, enabled the creation of Type I and Type II crack mechanisms, achieving an ultra-high gauge factor (GF) of up to 673. Furthermore, by introducing a UV picosecond laser at the PI/fluorinated ethylene propylene (FEP) bilayer interface, researchers also achieved in situ growth of fluorine-doped graphene, endowing the breathable electrodes with excellent superhydrophobicity and chemical stability [100].

In addition to carbon-based structures, laser direct writing can also be combined with chemical plating to achieve high-resolution metal patterning. Ji et al. proposed a three-step strategy of “pre-deposition-selective transfer-chemical plating”, which enabled the fabrication of high-resolution (~20 μm) copper patterns on porous, breathable substrates such as textiles and wood, demonstrating excellent mechanical interlocking and bending durability (8000 cycles without failure) [101]. Electrochemical deposition provides another route for constructing metal, oxide, and metal–organic framework coatings, with the final morphology controlled by the deposition mode and reaction conditions [102,103]. These techniques are suitable for direct metallization and interface-specific functionalization, but multistep processing, chemical residues, and process uniformity may limit manufacturing yield.

Chemical fabrication methods not only provide electrodes but also serve as sensing interfaces. In a systematic review, Qiao et al. pointed out that graphene can be integrated into various fields through laser direct writing and reduction processes, including mechanical, acoustic, electrophysiological, and fluid sensing [104]. To address potential structural instability issues that sensors may encounter in humid or biological environments, researchers have combined chemical construction with in situ polymerization to enhance device durability. For example, allows poly(vinyl alcohol) (PVA) membranes to retain their morphology and mechanical strength in aqueous environments [105]. This approach is useful when the conductive layer must be formed together with, or strongly bonded to, the substrate rather than deposited as a separate film.

In summary, chemical construction methods are therefore advantageous for customized patterns, porous substrates, and direct growth of conductive networks. Laser writing offers programmable patterning, plating provides high-conductivity metallic pathways, and in situ polymerization improves interfacial stabilization. Although these methods still face challenges such as high-temperature substrate damage or residues from wet chemical processes, with the optimization of laser scanning strategies and improvements in precision process control technologies, the chemical construction approach will become a key pathway for advancing breathable electronics toward industrial-scale production.

#### 2.3.4. Comparative Evaluation and Application Scenarios

The conductive-pathway strategies in Table 3 offer complementary advantages. Intrinsic conductive materials provide material-level flexibility and functional integration; physical methods enable precise conductor placement and interface control; and chemical methods allow direct, programmable patterning on specific substrates. Table 3 serves as a comparative reference rather than a direct performance ranking, since conductivity, sheet resistance, and interfacial impedance represent different aspects of electrical performance. Strategy selection should therefore consider electrical properties together with strain tolerance, interface stability, processing complexity, and scalability. Intrinsic materials suit compliant integrated devices, physical methods suit patterned or multilayer systems, and chemical methods suit substrate-specific conductive networks.

**Table 3:** Overview of the Conductivity, Advantages and Disadvantages of Various Conductivization Methods.

Design Dimension	Construction Strategy	Electrical Conductivity	Advantages	Disadvantages	Refs.
Intrinsically Conductive Materials	PEDOT:PSS + Plasticizer	>1000 S/cm	Excellent biocompatibility; compatible with solution processing	Resistance increases significantly under high strain	[85]
	n-type Polymer + H-bond Plasticization	2265 S/cm	Intrinsically stretchable; high electrical stability	Highly sensitive to ambient humidity	[28]
	ICP-coated Microneedle Arrays	Moderate	Enables minimally invasive breathable sensing	Low absolute electrical conductivity	[106]
Physical Construction Methods	Magnetron Sputtered ZnO Interface	Interfacial impedance decreased by 99%	Conformal interfacial contact; strong adhesion	Film brittleness; prone to micro-cracking	[92]
	AgNWs Network + Dry Transfer	10 Ω/sq	Ultra-high conductivity; good air permeability	Oxidation risk of metallic nanowires	[98]
	Bilayer Polymer Transfer Lamination	550.2 S/cm	Enables vacuum-free full solution processing	Process highly sensitive to thickness/reflectivity	[30]
Chemical Construction Methods	LIG	5–25 S/cm	One-step patterning; porous structure promotes breathability	Laser-induced high-temperature damage to substrate	[107]
	LIBT + Electroless Copper Plating	~2.0 × 10^−8^ Ω·m	Near-bulk metallic conductivity; robust interface	Complex multi-step process; limited yield	[101]
	Laser-Induced F-LIG	28 Ω/sq	Superhydrophobic; resistant to liquid erosion	Relatively thick; limited ultra-flexibility	[100]

## 3. Breathable Discrete Components

While stretchable substrates and breathable designs provide the foundation for physiological comfort, the realization of fully functional electronic systems relies heavily on high-performance discrete components. These fundamental units must seamlessly integrate electrical conductivity, mechanical deformability, and gas permeability into constrained geometries. This chapter systematically discusses the progress of key discrete components in breathable electronic systems, focusing primarily on two major categories: breathable interconnect wires and electrodes, which analyze conductivity, strain stability, and breathability under deformation, as well as breathable energy devices, which evaluate the performance, electrochemical stability, and gas-exchange capabilities of wearable power sources.

### 3.1. Breathable Interconnect Wires and Electrodes

In wearable breathable electronic systems, interconnect wires and electrodes play a crucial role in signal transmission, energy transport, and device coupling. However, traditional metal films or dense conductive layers often combine high electrical conductivity with low breathability, when worn against the skin for extended periods, they can impede water vapor exchange at the skin surface, leading to issues, such as sweat accumulation, skin irritation, and signal drift during physical activity [108]. Consequently, creating open pores via nanonets, porous fibers or micro- and nano-patterns to balance electrical conductivity and breathability has become a core design strategy [19].

From the perspective of material and structural design, breathable interconnect wires and electrodes can generally be categorized into three representative strategies.

**Figure 5:**
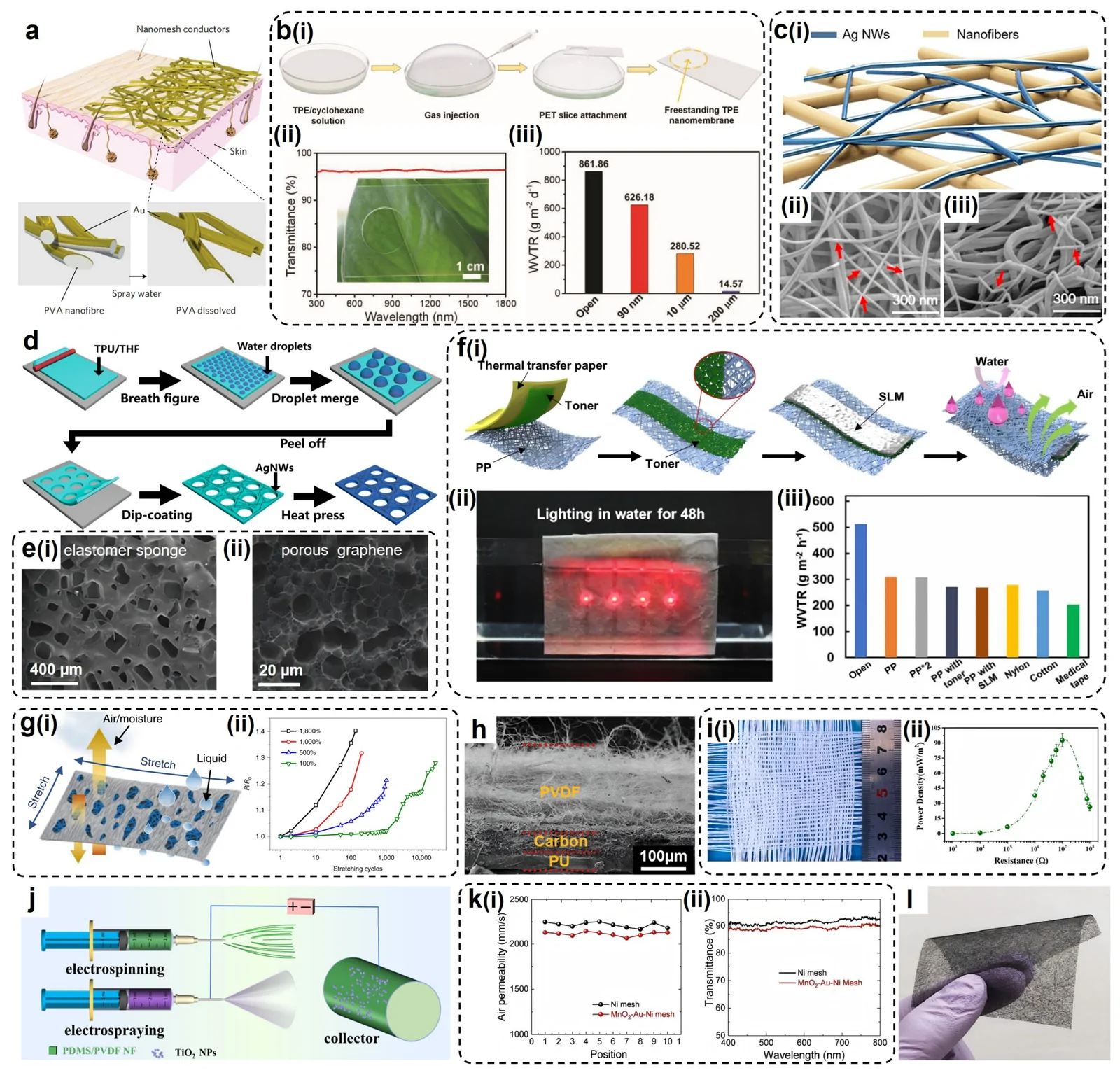
Material Systems and Fabrication Principles of Breathable Functional Components. (**a**) Substrate-free conductive nano-mesh Au electrode. Adapted from Ref. [32]. Copyright 2017, Springer Nature. (**b**) Thermoplastic elastic film: (**i**) bubble-blowing process, (**ii**) light transmittance characterization, (**iii**) WVP. Adapted from Ref. [109]. Copyright 2020, WILEY. (**c**) AgNWs@polyamide fiber: (**i**) schematic diagram of structure, (**ii**, **iii**) SEM images of AgNWs and nanowires. Adapted from Ref. [33]. Copyright 2018, American Chemical Society. (**d**) Steps for constructing the breathing pattern method. Adapted from Ref. [7]. Copyright 2020, American Chemical Society. (**e**) LIG SEM images of silicone sponge: (**i**) sugar-templated sponge, (**ii**) graphene. Adapted from Ref. [110]. Copyright 2018, WILEY. (**f**) SLM printing process: (**i**) preparation process, (**ii**) image of normal operation underwater, (**iii**) achievable water vapor flux of 311.1 g·m^−2^·h^−1^. Adapted from Ref. [111]. Copyright 2024, Springer Nature. (**g**) Pre-stretching to activate LM conductor permeability: (**i**) schematic diagram of the structure, (**ii**) diagram showing adaptive 1800% omnidirectional strain. Adapted from Ref. [112]. Copyright 2021, Springer Nature. (**h**) SEM characterization of the all-fiber electronic skin. Adapted from Ref. [34]. Copyright 2020, WILEY. (**i**) core–shell yarn structure TENG: (**i**) photograph, (**ii**) instantaneous power density plot. Adapted from Ref. [118]. Copyright 2021, Elsevier. (**j**) Schematic diagram of the preparation of porous fiber membranes via electrospinning. Adapted from Ref. [120]. Copyright 2023, ACS. (**k**) Performance demonstration of the all-solid-state supercapacitor: (**i**) air flux of up to 45.4 mm s^−1^, (**ii**) high light transmittance. Adapted from Ref. [117]. Copyright 2019, Elsevier. (**l**) Photograph of a 3D supercapacitor assembled from a thin mesh of activated carbon fibers. Adapted from Ref. [122]. Copyright 2018, WILEY.

The first category comprises designs based on nano-meshes or ultra-thin open structures, which achieve a virtually ‘invisible’ skin interface through extremely low areal density and open pores. For example, Miyamoto et al. [32] constructed a substrate-free conductive nanomesh electrode (Figure 5a) by using electrospun PVA nanofibers as a sacrificial template and depositing a thin layer of Au upon it. This structure relies on the open channels formed by the fiber network to allow free exchange of gas and water vapor, whilst retaining its lightweight and stretchable characteristics. It significantly reduces the risk of inflammation under continuous one-week wear conditions and supports touch, temperature and pressure sensing, as well as EMG recording. Yang et al. [109] employed a simple bubble-blowing method to fabricate an ultra-thin thermoplastic elastomer film with a thickness of just 150 nm (Figure 5b(i)) and constructed a conductive layer on top of it. Whilst maintaining a light transmittance of 83% (Figure 5b(ii)), the WVTR reached 580.18 g m^−2^ d^−1^ (Figure 5b(iii)), demonstrating the enhanced breathability achieved through extreme thinning. A common feature of these strategies is that breathability does not rely entirely on the pores themselves, but rather on shortening the water vapor diffusion path through extremely low thickness, open networks or non-continuous substrate structures. However, their ultra-thin structures typically still present challenges in terms of scale-up, operational stability and packaging reliability.

The second category of strategies involves conductive composite structures supported by fiber scaffolds or porous membranes. These methods typically incorporate conductive components into natural or synthetic fiber networks, establishing stable conductive pathways whilst preserving the original porosity. Fan et al. [33] embedded AgNWs into a polyamide nanofiber scaffold and, via vacuum filtration, produced an epidermal electrode that combines transparency, conductivity and breathability (Figure 5c). This achieved a light transmittance of 84.9% and a sheet resistance of 8.2 Ω/sq. Furthermore, after 3000 bending cycles or 2500 saltwater immersions, the rate of change in resistance was less than 0.1% and 1.5%. Zhou et al. [7] utilized a breathable patterning method to fabricate porous TPU (Figure 5d). Following dip-coating with AgNWs, they achieved a sheet resistance of 7.3 Ω/sq and a WVTR of 23 mg cm^−2^ h^−1^, striking a balance between breathability and low skin impedance. This approach effectively addresses the issue of conductive pathways fracturing under deformation by deeply coupling the process (vacuum filtration/breathable patterning) with the porous substrate. Compared to ultra-thin continuous films, such structures typically exhibit better mechanical robustness and wear resistance. However, patterning resolution, batch consistency, and long-term abrasion resistance remain key issues requiring further optimization.

The third category of strategies involves designs based on porous conductive carbon materials or direct patterning processes, aimed at low-cost manufacturing and multifunctional integration, representative methods include LIG and semi-liquid metal (SLM) printing. Sun et al. [110] fabricated laser-patterned porous graphene on a sugar-templated silicone sponge, creating a multifunctional, breathable, skin-adherent circuit that serves as an electrode, sensor and heating unit. Its water vapor flux reaches 18 mg cm^−2^ h^−1^, which is 18 times that of non-porous silicone rubber. Its breathability stems from two mechanisms (Figure 5e) on the one hand, the sugar-templated sponge itself provides continuous open channels. On the other hand, LIG forms a conductive network in situ on the pore walls, enabling the structure to perform electrical functions without the need for an additional densifying coating. Compared with traditional deposition-based electrodes, this approach offers the advantages of a simple process, lower cost and ease of patterning. However, inter-island contact resistance, conductive uniformity and the density of complex interconnections remain limited by the degree of material transformation and the precision of laser processing. Furthermore, Guo et al. [111] proposed a SLM printing process, combined with thermal transfer, to enable the rapid fabrication of multi-layer breathable and waterproof circuits (Figure 5f(i)). The resulting devices achieved a water vapor flux of 311.1 g m^−2^ h^−1^ (Figure 5f(iii)) and remained functional underwater (Figure 5f(ii)), with a wash resistance exceeding 120 min. Ma et al. [112] further utilized a composite of LM and Styrene-Butadiene-Styrene (SBS) fiber felt, through pre-stretching (Figure 5g(i)), they enabled the LM to self-organize into a transverse mesh and longitudinal pleated conductors to activate permeability, ultimately constructing a conductive fiber felt that combines ultra-high stretchability with breathability. This achieved a conductivity of 1.8 × 10^6^ S m^−1^ and was capable of adaptively withstanding 1800% omnidirectional strain (Figure 5g(ii)), demonstrating the immense potential of LM conductors in highly stretchable, breathable integrated systems.

Overall, the design of breathable interconnect wires and electrodes essentially involves finding the optimal balance between conductivity, breathability and mechanical stability: nano-meshes and ultrathin films are suitable for lightweight sensing, fiber scaffolds are suited to the durability required for long-term service, whilst laser and printing processes facilitate rapid prototyping. In terms of performance metrics, these studies typically focus on evaluating sheet resistance, resistance stability, breathability, and signal retention under cyclic deformation. For example, nanomesh and porous fiber structures often achieve significantly reduced interfacial impedance whilst maintaining low sheet resistance, whereas LMs and LIG place greater emphasis on conductive continuity under complex deformation and in multi-layer integration [7,32,110,111,112]. However, most current research remains focused on the performance validation stage for individual devices. Systematic challenges specific to long-term wear, such as interface aging following exposure to sweat, synergistic failure of conductive and encapsulation layers, and high-density integration with multi-channel interconnections, still require further resolution [113,114].

### 3.2. Breathable Energy Devices

Energy modules for wearable electronics must not only provide a stable power supply, but also ensure comfort during long-term contact with the skin. Traditional batteries or energy storage devices typically rely on hermetically sealed packaging and rigid current collectors, making it difficult to meet the need for the human skin to “breathe” continuously. Consequently, breathable power devices have gradually become a research priority [115,116,117]. Existing work has primarily followed two avenues: one involves self-powered devices based on triboelectric nanogenerators (TENGs), whilst the other focuses on flexible energy storage devices based on supercapacitors.

In the field of self-powered devices, breathability is often achieved through porous, fibrous or fabric-like structures. Li et al. [34] constructed a fully fibrous electronic skin using scalable electrospinning, integrating a porous elastic substrate, a triboelectric layer and electrodes into a single unit (Figure 5h). Whilst retaining fabric-level breathability, they achieved the coupling of pressure sensing and energy output, with a WVTR of 10.26 kg m^−2^ d^−1^, demonstrating the immense potential of fiber-based frameworks in balancing comfort with sensing and energy harvesting. Guan et al. [118] further employed a core–shell yarn structure (Figure 5i(i)), coating the surface of stainless steel yarns with polyamide 66 (PA66) and poly(vinylidene fluoride-trifluoroethylene) (PVDF-TrFE) and weaving them into a TENG. Whilst maintaining the fabric’s porosity, they achieved an instantaneous power density of 93 mW m^−2^ (Figure 5i(ii)), effectively alleviating the loss of breathability caused by traditional coatings. The power-generating fabric developed by Qiu et al. achieved a balance between high breathability and mechanical durability through a fully fibrous structure, demonstrating that the fabric form can not only ensure wearing comfort, but also directly convert human kinetic energy without the need for complex encapsulation [119]. In addition to fabric structures, open-pore coatings and porous nanofiber membranes are also effective means of enhancing breathability. Sun et al. [120] prepared a PVDF/PDMS/TiO_2_ porous fiber membrane (Figure 5j) via electrospinning and spray-drying. By utilizing TiO_2_ to enhance dielectric properties and surface roughness, they achieved simultaneous optimization of multifunctional properties including water repellency, breathability and enhanced power output. Zheng et al., on the other hand, utilized a porous nitrocellulose membrane to construct a water-repellent, breathable TENG, demonstrating that natural/derived cellulose materials can also serve as lightweight, wearable friction layers [121]. These studies indicate that the essence of TENGs lies not simply in ‘making them porous’, but in enhancing contact efficiency, reducing sweat accumulation and preserving air exchange pathways through pore structure design.

Compared with power-generating devices, the design of breathable supercapacitors relies more heavily on 3D porous electrodes to balance ion diffusion and storage capacity. Xu et al. [117] used an ultra-thin Ni metal mesh as the current collector and deposited MnO_2_ (Figure 5k) to construct an all-solid-state supercapacitor that achieved an air flux of up to 45.4 mm s^−1^ under a pressure difference of 100 Pa, while exhibiting extremely high light transmittance and folding stability. with a capacity retention rate exceeding 90% even after 10,000 cycles, it demonstrated the high compatibility between the conductive properties and air permeability of the open-mesh metal mesh. Shin et al. [122], on the other hand, directly assembled a 3D supercapacitor using a thin activated carbon fiber mesh (Figure 5l), utilizing the natural porosity of the fiber network to enhance electrolyte wetting and ion diffusion efficiency. The device was able to complete 10,000 charge–discharge cycles even under folding and compression deformation, with a capacity retention rate exceeding 86.6%. Wang et al. laminated gold foil onto polyester fabric and loaded it with polypyrrole to produce a sewable energy fabric, demonstrating the concept of transforming traditional textile substrates into energy storage platforms [123]. Such work illustrates that breathable supercapacitors do not come at the expense of electrochemical performance. Rather, structural optimization enables the synergistic enhancement of electrical performance and comfort. The key lies in striking a balance between the open electrode structure and the electrochemically active layer: the greater the porosity, the better the comfort and ionic transport. However, this can also limit the active material loading and volumetric energy density. To further address these capacity limitations, the integration of binary transition metal oxides (BTMOs) and binary metal oxides (BMOs) into porous scaffolds presents a highly promising strategy. These advanced nanocomposites provide rich multivalent redox activities and structural robustness, which can significantly enhance the specific capacitance and long-term cycling stability of breathable supercapacitors without sacrificing their gas-exchange capabilities [124].

Overall, technological innovation in breathable energy devices primarily involves the following: first, introducing open pores through structural design to achieve a balance between breathability and mechanical properties, thereby enhancing energy density whilst maintaining wearing comfort; second, through the synergistic design of materials and scaffolds, advancing energy devices towards self-powered, washable, and fabric-integrated solutions [125,126]. Although the field has now made the leap from device prototypes to fabric integration, further challenges requiring breakthroughs in future research include enhancing long-term stability during wear, reducing the impact of humidity on output performance, and achieving modular integration with flexible circuits [127,128].

### 3.3. Comparative Evaluation and Application Scenarios

The breathable discrete components discussed in this chapter can be evaluated according to their functional role in the system. For interconnects and electrodes, the key criteria include electrical resistance, signal stability under deformation, breathability, interfacial impedance, environmental durability, and integration capability. For energy devices, additional criteria, such as power output, energy-storage capacity, cycling stability, humidity tolerance, and packaging requirements must be considered. Therefore, the performance of these two types of components should be compared within their respective functional contexts rather than by a single universal metric.

For breathable interconnects and electrodes, nanomeshes and ultrathin open structures minimize material coverage and interfacial obstruction, making them suitable for lightweight, transparent, and conformal electrophysiological electrodes. However, their limited structural thickness may compromise handling, packaging reliability, and large-area integration. Fiber-supported conductive composites generally provide better mechanical robustness and wear resistance because the conductive network is reinforced by a porous scaffold, and are therefore more attractive for textile-integrated and long-term wearable systems. Their performance, however, depends strongly on conductive-layer uniformity, interfacial adhesion, and resistance to abrasion, washing, and sweat exposure. Porous carbon and LM-based structures offer advantages in direct patterning, multifunctional integration, and large-strain operation, but may suffer from resistance nonuniformity, limited pattern resolution, inter-island contact resistance, or process-control challenges. Thus, nanomeshes and ultrathin structures are particularly suitable for low-profile sensing and transparent skin interfaces, fiber-supported networks are preferable for durable textile systems, and laser-patterned or LM architectures are promising for highly deformable, multifunctional circuits.

Breathable energy devices involve a different set of trade-offs. Fibrous and porous TENGs can simultaneously provide breathability, mechanical compliance, and self-powered signal generation, making them suitable for intermittent sensing, motion-triggered output, and energy-autonomous textile interfaces. Their electrical output, however, is highly dependent on contact area, excitation frequency, deformation amplitude, humidity, and surface condition, which can lead to substantial differences among reported power densities. Breathable supercapacitors provide more controllable energy storage and are better suited for energy buffering or short-term power supply, but increasing porosity may reduce active-material loading and volumetric energy density. In addition, electrolyte retention, encapsulation, and long-term stability under sweat and repeated deformation remain important limitations. Accordingly, TENGs are more appropriate for low-power, intermittent operation, whereas breathable supercapacitors are better suited to systems requiring energy accumulation and relatively stable output.

The differences among reported results should therefore be interpreted in relation to device architecture and operating conditions, rather than treated as direct contradictions. For electrodes, apparent differences in resistance stability or stretchability may arise from variations in conductive-network morphology, substrate constraint, strain mode, and cycling protocol. For energy devices, output and storage performance are additionally affected by mechanical excitation, contact conditions, electrolyte configuration, humidity, and packaging. Table 4 in this chapter summarize the reported performance of individual studies, while the comparisons above identify the relative strengths, limitations, and application scenarios of each strategy. From a system-level perspective, future breathable electronics should combine low-impedance and mechanically stable interconnects with energy modules whose power output, storage capacity, moisture tolerance, and packaging requirements are compatible with the target application. Manufacturing scalability will ultimately depend on the compatibility of these architectures with textile processing, large-area patterning, encapsulation, and reliable integration of multiple components.

**Table 4:** Characteristics of Breathable Discrete Components.

Functional Category	Strategy Category	Specific Technical Approach	Breathability Metric (WVTR/Flux)	Performance Metric (Conductivity/Energy)	Stability/Robustness	Refs.
Wires & Electrodes	Nanomesh/Ultra-thin	Au Nanomesh/TPE Film	>580 g/(m^2^·d)	Low Sheet Resistance	1-week wear (no irritation)	[32]
	Fiber Scaffold	AgNWs @ Polyamide Fiber	84.9% Transparency	8.2 Ω/sq	3000 bending cycles	[33]
		Porous TPU/AgNWs	23 mg/(cm^2^·h)	7.3 Ω/sq	Long-term wear-resistance	[7]
	Laser/Patterning	LIG Porous Graphene	18 mg/(cm^2^·h)	Joule heating/ECG	Sweat/corrosion resistant	[110]
		LM (EGaIn)	311 g/(m^2^·h)	1.8 × 10^6^ S/m	1800% stretchability	[111]
Energy Devices	TENGs	All-fiber Membrane	10.26 kg/(m^2^·d)	0.72 Wd/m^2^	50% strain stability	[34]
		Yarn-weaving/Textile	High air permeability	93 mW/m^2^	Washable/Wearable	[118]
	Supercapacitors	Nitrocellulose membrane	Waterproof & Breathable	78.3 mW/m^2^	High humidity stability	[121]
		Metal mesh/Activated Carbon	45.4 mm/s	>90% Capacitance retention	10,000 cycles	[117]
		Gold leaf/PPy Textile	Textile-grade breathability	Excellent specific capacity	Multi-axial deformation	[123]

## 4. Practical Applications of Breathable Electronics

To bridge the gap between fundamental electronic mechanisms and practical biomedical applications, this chapter reviews the cutting-edge developments of breathable electronics. While the following subsections are organized by technical sensing mechanisms (mechanical, electrophysiological, and biochemical) to maintain structural coherence with the previous chapters, these technologies are fundamentally developed to serve specific areas of human life. As summarized in Table 5, mechanical sensors are extensively applied in the monitoring of the human respiratory system and limb rehabilitation; electrophysiological sensors are the cornerstones of cardiovascular and neurological health management; and biosensors play a vital role in metabolic monitoring and wound care. In the following sections, the discussion of each sensor type will explicitly highlight its target human life area.

### 4.1. Mechanical Sensors

Mechanical sensing is one of the most representative applications of breathable electronics. Conventional flexible sensors are commonly supported by dense polymer films, which restrict heat and moisture transport during prolonged skin contact. By contrast, nanofibrous membranes, porous elastomers, textiles, sponges, and nanomeshes preserve air and water-vapor exchange while introducing deformable interfaces that amplify electromechanical responses. In this section, normal compressive loading is discussed as pressure sensing, whereas tensile, bending, and torsional deformation is categorized as strain sensing.

#### 4.1.1. Pressure Sensing

In the context of specific human life areas, breathable pressure sensors are particularly essential for the monitoring of the human respiratory system, where they can capture subtle chest movements. Breathable pressure sensors predominantly leverage piezoresistive or capacitive transduction. In these systems, porous microstructures serve a dual function: providing passive ventilation pathways and dynamically modulating conductive contact areas, quantum tunneling distances, capacitive electrode spacing, and dielectric polarization under mechanical loads.

To mitigate moisture-induced signal instability in humid microenvironments, hydrophobic and porous dielectric architectures have been extensively explored. Wang et al. developed a washable capacitive textile that detected pressures as low as 20 Pa with 30 ms response and recovery at 0.2 Hz, remained stable up to 70% relative humidity (RH), and survived more than 10 washes [129]. The device detected pressures as low as 20 Pa with response and recovery times of approximately 30 ms, while maintaining stable outputs at relative humidities of up to 70% and after repeated washing. This work established that moisture resistance and washability are essential for practical breathable pressure sensors, particularly when they are integrated into clothing for pulse, throat-motion, and limb-motion monitoring.

**Figure 6:**
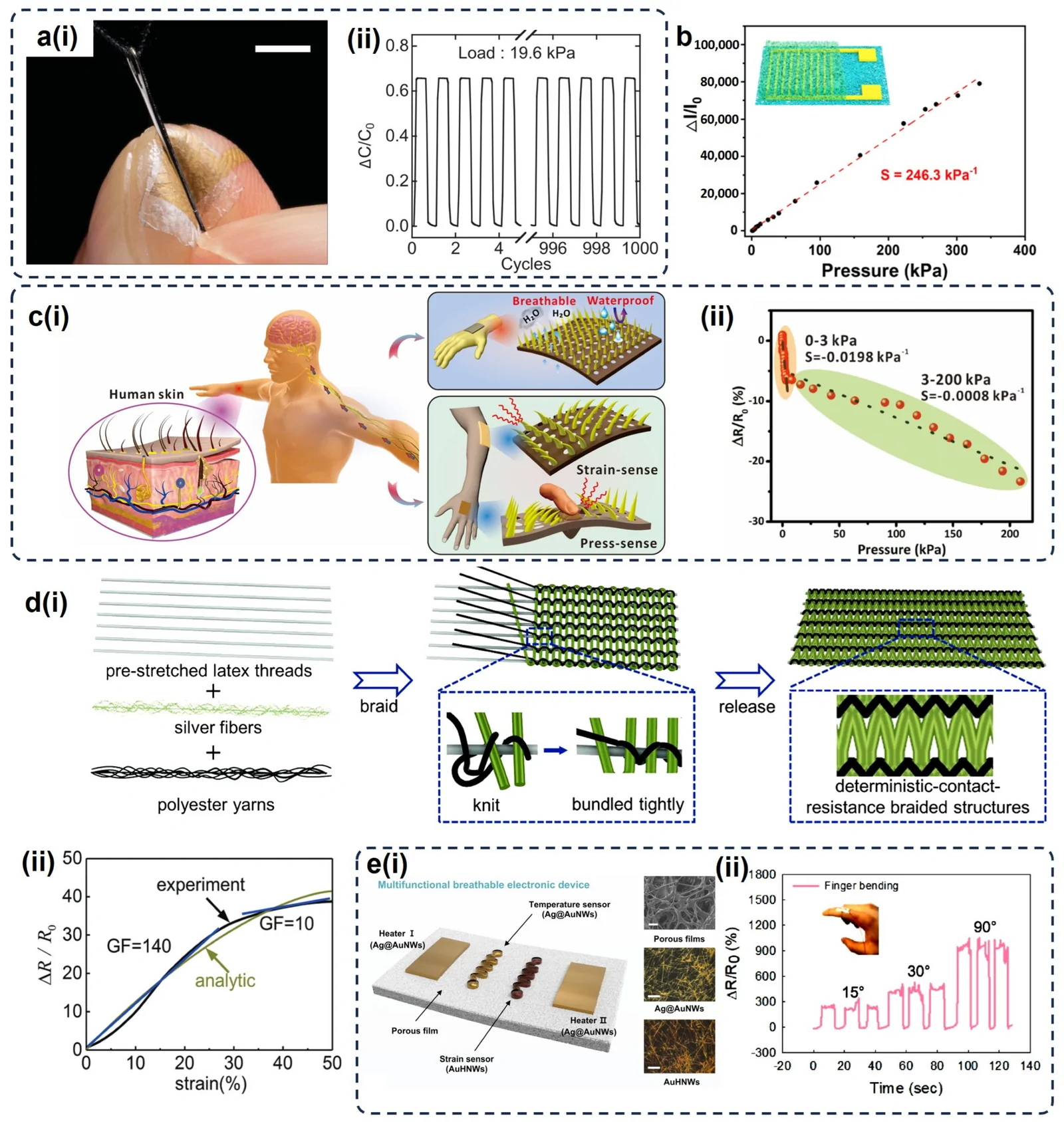
Various components and performances of breathable mechanical sensors. (**a**) The nanomesh pressure sensor: (**i**) Nanomesh pressure sensor attached to an index finger. Scale bar, 5 mm, (**ii**) Pressure sensitivity of the nanomesh sensor during 1000 cyclic pressure applications. Adapted from Ref. [36]. Copyright 2020, American Association for the Advancement of Science. (**b**) The sensing sensitivity of the bioinspired breathable electronic sensor with external pressure up to 333.3 kPa. Adapted from Ref. [132]. Copyright 2024, Chemical Engineering Journal. (**c**) Breathable and waterproof electronic skin with 3D architecture for pressure and strain sensing: (**i**) Schematic diagram of the composition and sensing mechanism of the human skin, (**ii**) Pressure-sensing performance of the electronic skin (e-skin) with a pressure from 0 to 200 kPa. Adapted from Ref. [133]. Copyright 2022, American Chemical Society. (**d**) Stretchable strain sensors based on deterministic-contact-resistance braided structures: (**i**) The schematics of fabricating the strain sensor, (**ii**) Experimental and theoretical comparisons of the relationship between relative resistance and applied sensor strain. Adapted from Ref. [37]. Copyright 2022, WILEY. (**e**) The multifunctional breathable wearable device: (**i**) Materials and structures, (**ii**) Real-time monitoring of finger bending with strain sensors. Adapted from Ref. [138]. Copyright 2023, Chemical Engineering Journal.

Beyond passive thermal-moisture comfort, minimizing mechanical restraint and sensory interference at sensitive skin interfaces represents a critical paradigm shift. Lee et al. reported an ultrathin capacitive nanomesh pressure sensor that could be attached directly to the fingertip (Figure 6a(i)) without disrupting natural grip and achieved a sensitivity of 0.141 kPa^−1^ below 1 kPa [36]. Its capacitance changed by less than 0.15% over 1000 cycles at 19.6 kPa (Figure 6a(ii)), but sensitivity fell to 0.010 kPa^−1^ above 10 kPa. This contrast shows that very high sensitivity at low-pressure range does not guarantee consistent performance across the full tactile range, even though the nanomesh preserved natural manipulation.

Porous conductive networks provide a second route to high sensitivity. Chao et al. assembled a Ti_3_C_2_T_x_ MXene-coated silk-fibroin nanofiber membrane with printed interdigitated electrodes [130]. The interconnected fibrous pores supported vapor transport, whereas compression caused large variations in interfacial contact resistance, producing sensitivities of 298.4 and 171.9 kPa^−1^ in two pressure regimes, response/recovery times of 7/16 ms, and stable operation over 10,000 cycles. By contrast, a pad-dried MXene/cellulose textile reached a lower sensitivity of 28.723 kPa^−1^ but retained an air permeability of 954.9 mm·s^−1^ [131]. This comparison reveals a persistent trade-off between maximum sensitivity, manufacturability and inherent permeability. It also shows that a single sensitivity value cannot identify the best practical design without the pressure range, fabrication route and transport metric.

Recent advances have propelled breathable pressure sensors beyond single-parameter detection toward ultra-broad operational ranges and intrinsic multimodal decoupling. An ant-nest MXene sponge (Figure 6b) reached 246.3 kPa^−1^ over 333.3 kPa and remained stable for approximately 20,000 cycles [132]. Its sensitivity reached 246.3 kPa^−1^, the sensing range extended to 333.3 kPa, and stable operation was maintained for approximately 20,000 cycles, enabling both subtle physiological sensing and large-motion detection. In parallel, Lei et al. developed a breathable and waterproof electronic skin with 3D conductive microcilia (Figure 6c) [133]. Pressure increased the number of conductive contacts and reduced resistance, whereas tensile strain separated conductive components and increased resistance. The opposite signal directions enabled pressure–strain discrimination within a single device. The sensor showed a pressure sensitivity of −0.0198 kPa^−1^ over 0–3 kPa, a strain gauge factor of 7.747 over 0–80%, a water-vapor evaporation rate of 6.7397 mg cm^−2^ h^−1^, and stable strain responses over 10,000 cycles. Thus, intrinsic decoupling is feasible, but often at the cost of sensitivity, structural simplicity or evidence for large-scale manufacturing.

#### 4.1.2. Strain Sensing

Targeting specific human life areas such as limb rehabilitation and full-body motion tracking, breathable strain sensors detect tensile, flexural, or torsional deformation via dynamic microstructural evolutions. Breathable strain sensors detect tensile, flexural, or torsional deformation via dynamic microstructural evolutions, including microcrack propagation, fiber-to-fiber contact modulation, conductive network geometry shifting, and electrode displacement. Particularly in polymer composites utilizing carbon nanotubes and graphene, these geometric shifts govern the piezoresistive response by dynamically reconfiguring the percolation network. The resulting exponential variations in interparticle tunneling resistance provide the fundamental mechanism for transducing mechanical strain into highly sensitive electrical signals [134]. Compared with pressure sensors, strain gauges must accommodate larger deformations while maintaining sufficient resolution to detect subtle skin motions. Accordingly, research has focused on optimizing the trade-off among sensing range, gauge factor, cyclic repeatability, and mechanical imperceptibility.

All-fabric and nanomesh strain gauges reduce mechanical constraint during large body movements. Ho et al. stacked blow-spun PVDF-HFP dielectric nanofibers with SWCNT/PVDF-HFP conductive nanofibers to form an all-fabric capacitive strain sensor [135]. The open fibrous structure exhibited a gauge factor above 130, a minimum detectable strain of 0.03%, and stable responses over 1000 cycles. Further minimizing mechanical constraint on natural skin, a 430 nm Au/PU-PDMS nanomesh achieved gauge factors of 7.26–46.3, linear sensing up to 60%, 5000 cycles at 60% strain and a 5.3% drift after 12 h static stretch at 40% [136]. The 12 h static-stretch test characterizes short-term drift, not long-term creep or service life. The nanomesh, therefore provides the best mechanical imperceptibility, whereas the textile route offers a larger and more robust sensing range. The reported linear ranges varied with mesh geometry, however, and the static drift result cannot be directly extrapolated to repeated washing or long-term wear.

To overcome the severe hysteresis and structural randomness inherent to disorganized nanofiber mats, advanced textile engineering introduces deterministic geometric constraints. Li et al. developed a deterministic-contact-resistance braided sensor in which polyester yarns constrained silver fibers and elastic latex threads (Figure 6d(i)) [37]. The controlled contact geometry produced a gauge factor of up to 140, a sensing range of 50% (Figure 6d(ii)), and a repeatability error of 3.74%, together with washability and tolerance to local defects. In contrast to this strategy of suppressing structural uncertainty, Yang et al. exploited the complex behavior of textile networks [137]. Their graphene-coated textile sensor operated over strains approaching 120% and exhibited negative differential resistance caused primarily by the relative displacement and rearrangement of fibers within the bundles. This neural-spike-like response was further used for strain-threshold detection and near-sensor processing, indicating that breathable textile mechanics can provide functional signal transformation rather than simple monotonic sensing.

Finally, breathable strain sensors are increasingly being integrated into multifunctional healthcare systems. Hong et al. deposited Au hollow nanowires on a porous SEBS substrate with a modulus of 0.3 MPa (Figure 6e(i)) [138]. After device integration, the water-vapor transmission rate remained 2428.9 ± 9.1 g m^−2^ day^−1^, while the strain sensor produced a relative resistance change of 19.07 at 80% strain and showed limited temperature cross-sensitivity, but long-term wear, washing and aging were not independently validated. This combination enabled reliable monitoring of finger (Figure 6e(ii)), facial, neck, and leg movements within a shared strain-temperature-heating platform.

Among mechanical sensors, the most critical degradation modes are microcrack growth, conductive-network fatigue, fiber pull-out, washing-induced abrasion, and interfacial delamination. These processes increase hysteresis and signal drift and may interrupt conductive pathways even when the substrate remains intact. Prevention should combine deterministic or braided architectures, mechanically reinforced junctions, robust conductor-substrate adhesion, low-modulus elastic interconnects, and breathable encapsulation; wash and cyclic-load tests should also be performed together rather than as separate tests.

Across mechanical designs, the most effective strategy depends on the required pressure or strain regime rather than on sensitivity alone. Hierarchical porous contacts and polarizable porous dielectrics provide the highest low-pressure sensitivity, while deterministic braided and textile structures currently offer the best balance of repeatability, breathability, washability and scalable manufacturing. Ultrathin nanomeshes remain superior for imperceptible mounting and subtle skin motion, but their cleanroom fabrication and susceptibility to handling damage or delamination limit practical scale-up. Random microcrack and weakly constrained fibrous networks are the most failure-prone because progressive fiber slip and unstable contacts cause hysteresis and drift, whereas excessive porosity can collapse or interrupt conductive pathways under repeated loading. The central trade-offs are sensitivity versus linear range, breathability versus mechanical and electrical integrity, and conformability versus durability. For pressure sensing, hierarchical porous interfaces are most promising for low-pressure monitoring; for whole-body motion, braided textiles are the most credible scalable route; and nanomeshes are best reserved for applications in which minimal mechanical interference outweighs manufacturing cost.

### 4.2. Electrophysiological Sensors

Electrophysiological signals originate from ionic currents generated by excitable tissues and are converted into electronic signals at the skin-electrode interface. ECG, EMG, EEG, and EOG provide information on cardiac activity, neuromuscular function, brain dynamics, and eye movement. Because these signals are weak and susceptible to motion, dehydration, and interfacial impedance fluctuations, reliable acquisition requires intimate and stable skin contact.

Conventional Ag/AgCl gel electrodes provide low initial impedance but may dry out, trap perspiration, irritate the skin, and delaminate during long-term wear. Breathable electronics address this limitation through porous films, nanofiber networks, textiles, and ultrathin architectures that preserve heat and moisture exchange while maintaining electrical continuity and mechanical compliance [20,139].

#### 4.2.1. ECG Sensing

**Figure 7:**
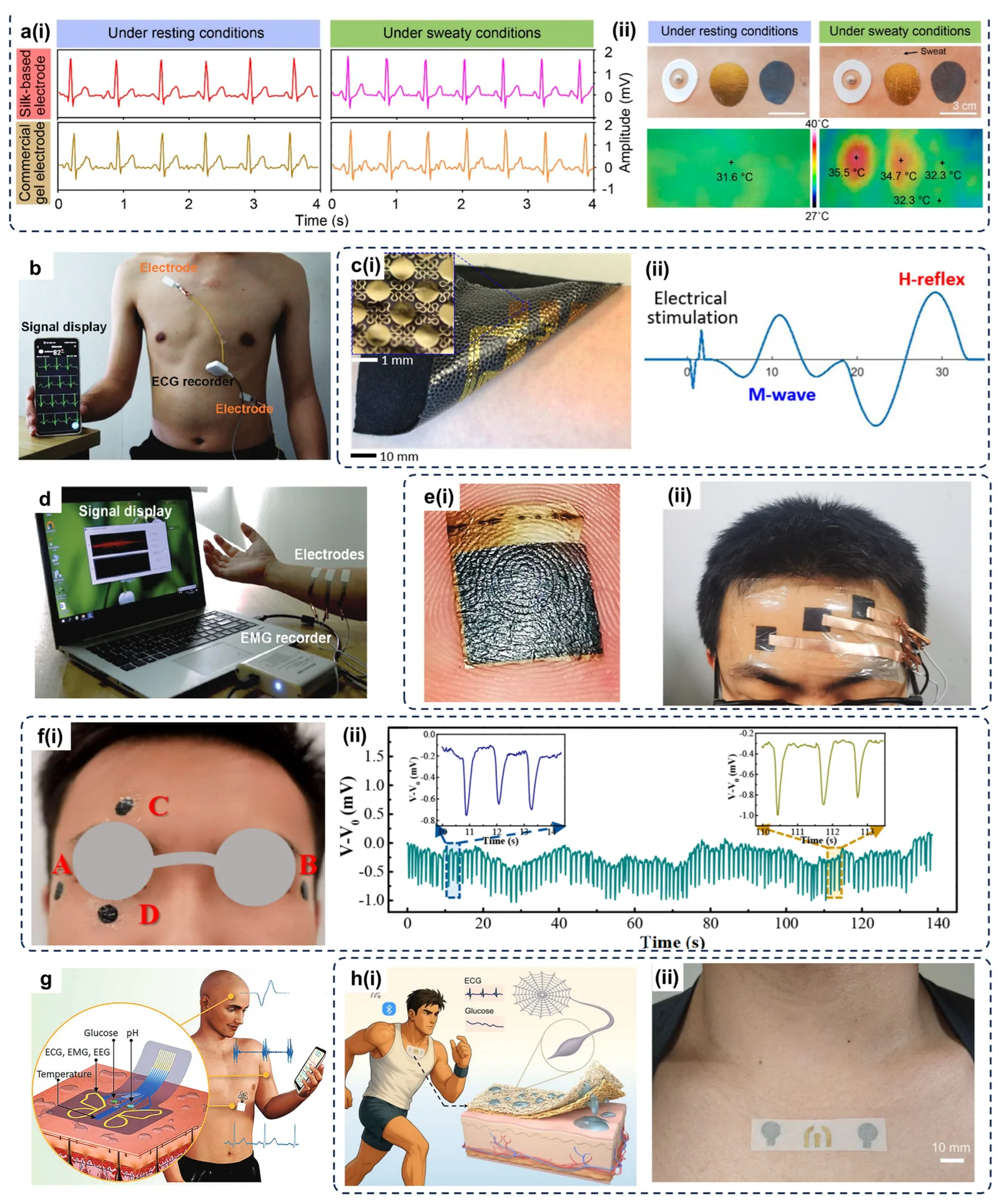
Breathable electronic devices applied to the monitoring of various physiological signals. (**a**) Silk-based electrodes with sweat tolerance: (**i**) ECG signals recorded by silk-based and commercial electrodes before and after exercise, (**ii**) The good thermal transmission of silk-based electrodes. Adapted from Ref. [140]. Copyright 2021, American Chemical Society. (**b**) Janus textile electrodes for real-time detection of ECG signals. Adapted from Ref. [141]. Copyright 2022, WILEY. (**c**) The L-EES: (**i**) Photo of a fabricated L-EES, gently placed on the skin (forearm), along with the enlarged view of a single electrode in the array, (**ii**) Representative EMG response following transcutaneous electrical stimulation of the median nerve. Adapted from Ref. [143]. Copyright 2020, Elsevier. (**d**) Janus textile electrodes for real-time detection of EMG signals. Adapted from Ref. [141]. Copyright 2022, WILEY. (**e**) Graphene Nanomesh e-skin. (**i**) The LSG/PU nanomesh on finger with clear fingerprint morphology (Yellow part is GO/PU nanomesh. Black part is LSG/PU nanomesh), (**ii**) EEG e-skin attached to the forehead of the tester. Adapted from Ref. [144]. Copyright 2021, WILEY. (**f**) Honeycomb graphene electrode for EOG monitoring. (**i**) Schematic diagram of the potential difference between the retina and cornea of the eyeball, (**ii**) Continuous eye-blink signal acquisition for more than 2 min. Adapted from Ref. [146]. Copyright 2022, American Chemical Society. (**g**) Monitoring system mounted on the chest for ECG/EMG/EEG monitoring with simultaneous detection of sweat glucose, pH, and temperature. Adapted from Ref. [147]. Copyright 2022, WILEY. (**h**) The spindle-structured nanomesh-based flexible system with directional sweat transport: (**i**) Schematic diagram of the multimodal monitoring system, (**ii**) The thin and breathable sensor placed on the chest. Adapted from Ref. [148]. Available under a CC-BY 4.0 license.

As a cornerstone for cardiovascular health management, ECG records cardiac depolarization and repolarization and is central to arrhythmia screening and continuous telemetry. ECG records cardiac depolarization and repolarization and is central to arrhythmia screening and continuous telemetry. Under sweating, baseline drift caused by interfacial instability can obscure the P, QRS, and T waves. Li et al. developed glycerol-plasticized silk/PEDOT:PSS electrodes with a stretchability above 250%, a WVTR of approximately 117 g m^−2^ h^−1^ at 37 °C, and an evaporative resistance of about 23 Pa m^2^ W^−1^ [140]. The electrodes retained recognizable ECG waveforms after exercise, avoided the local temperature rise observed with polymer-film and commercial gel electrodes, and supported recording for over 7 consecutive days (Figure 7a). Advancing from passive permeability to active sweat management, Yang et al. developed a Janus nanofiber electrode that transported sweat away within 5 s and limited ECG baseline deviation to 12% during sweating (Figure 7b) [141]. Tian et al. combined monolayer pores, Au microcracks and wet adhesion to maintain stable impedance after 1000 cycles at 30% strain and improved the crossover frequency to 4 kHz after sweating [142]. These developments delineate a clear paradigm shift from simple permeable substrates toward directionally sweat-pumping, wet-adhesive, and strain-insensitive biopotential interfaces.

#### 4.2.2. EMG Sensing

In the realm of neuromuscular health and motor rehabilitation, EMG quantifies motor-unit action potentials (MUAPs) to evaluate neuromuscular function, prosthetic control, muscle fatigue, and motor rehabilitation. Compared with localized ECG acquisition, EMG mapping demands spatially distributed, multichannel electrode arrays capable of adhering conformally across highly dynamic, heterogeneous muscular contours during intense movement. Kwon et al. developed a breathable large-area epidermal electronic system (L-EES) containing a 4 × 4 array of 16 Au nanomembrane electrodes (Figure 7c(i)) [143]. Its water-vapor transmission of 3.13 ± 0.18 g h^−1^ m^−2^ was comparable to clinical Tegaderm, while the array recorded M-waves and H-reflexes comparable to conventional electrodes (Figure 7c(ii)) and avoided the erythema produced by gel electrodes after 8 h. For exercise and sweating, the Janus electrode of Yang et al. exhibited an EMG signal-to-noise ratio (SNR) reduction of only 0.35 dB (Figure 7d), compared with 6.56 dB for Ag/AgCl electrodes, and its baseline deviation increased by merely 7% [141]. Likewise, the silk-based electrodes maintained high-quality EMG at approximately 100–150% elongation [140]. Thus, breathable EMG platforms are evolving from single-point dry electrodes toward conformal arrays and textile-integrated interfaces capable of mapping dynamic muscle activity. Multichannel mapping benefits most from a combination of spatial coverage, uniform skin contact and sweat removal; increasing channel count alone does not ensure better performance.

#### 4.2.3. EEG Sensing

Addressing the critical human life areas of neurological monitoring and human–machine interaction, EEG captures microvolt-scale cerebral activity and is relevant to sleep staging, fatigue detection, neurological diagnosis, and brain–computer interfaces. It is more vulnerable to interfacial impedance and motion because its signals are microvolt-scale. The extremely low amplitude of EEG signals renders them exceptionally vulnerable to interfacial impedance fluctuations and motion-induced capacitive noise, requiring ultrathin, ultra-conformable electrode architectures. Qiao et al. fabricated a laser-scribed graphene/PU nanomesh e-skin (Figure 7e(i)) with a thickness of approximately 15.6 μm and a mass of only 0.3 mg cm^−2^ [144]. Laser removal of insulating nanofibers reduced the interface impedance by roughly one order of magnitude, yielding values below those of gel electrodes at frequencies under 1 kHz. The electrode detected EEG (Figure 7e(ii)) and enabled five-level attention classification, with test accuracy improved to 48.7% using a modified ResNet18. Dong et al. subsequently introduced permeable SEBS-Ag-EGaIn textiles with 1032% elongation and a moisture permeability of 1895.49 g m^−2^ day^−1^ [145]. Besides a surface EMG SNR of 23.2 dB, the electrodes resolved the expected 8–12 Hz alpha rhythm and its attenuation upon eye opening. The key comparison is therefore not minimum thickness but whether low impedance, motion tolerance and breathability remain stable over repeated wear.

#### 4.2.4. EOG Sensing

EOG measures the standing corneo-retinal potential difference created by the metabolically active retinal pigment epithelium, enabling robust tracking of horizontal and vertical eye movements, blink dynamics, fatigue progression, and hands-free human–machine interaction (HMI). Xu et al. prepared ultrathin honeycomb graphene electrodes on a 45 μm breathable PU film (Figure 7f(i)) [146]. The electrodes recorded blink signals with an amplitude of approximately −0.68 mV and an SNR of 38.7 dB, remained stable for about 100 blinking cycles, (Figure 7f(ii)) and classified nine eye movements with an average accuracy of 92.6%. Their output also varied approximately linearly with gaze angle. A monolayer porous electrode with 0.17 N cm^−1^ wet adhesion continued recording after the gel reference delaminated under forehead sweating, increasing blink SNR from 11.16 to 18.04 [142]. These results support sweat tolerance and gesture recognition, but the short blink-cycle test does not establish multiday stability. Electrode placement, gaze angle and facial motion remain major sources of failure.

#### 4.2.5. Multimodal Integration

Contemporary wearable bioelectronics is rapidly expanding from single-channel biopotential acquisition toward integrated, multimodal diagnostic platforms that concurrently correlate electrophysiological signals with physical biomechanics and microfluidic biochemical biomarkers. Sun et al. integrated electrophysiological, hydration, temperature, and heating functions using porous laser-induced graphene on sugar-templated elastomer sponges [110]. The platform achieved a WVTR of approximately 18 mg cm^−2^ h^−1^, about 18 times that of nonporous elastomer, and showed only about 1% resistance increase after 1000 cycles at 500% strain.

As an important integration benchmark, Zahed et al. introduced a nonporous butterfly patch (Figure 7g) that combined ECG/EMG/EEG with sweat glucose, pH and temperature sensing, providing a functional benchmark but not evidence for breathable operation [147]. More recently, Wu et al. translated such multimodal functionality into a genuinely permeable platform by integrating wireless ECG and sweat-glucose monitoring with a spindle-structured directional sweat-pumping nanomesh (Figure 7h) [148]. The system provided a WVTR of 132.6 g m^−2^ h^−1^ and a directional liquid-transport capacity of 4.00 mL min^−1^ cm^−2^, approximately 1200 times the typical exercise sweat rate, enabling stable monitoring during one hour of treadmill activity. These advances establish breathability not merely as a comfort feature, but as a system-level requirement for preserving interfacial fidelity and enabling long-duration, multimodal physiological interpretation.

Across electrophysiological platforms, the most effective interface depends on the dominant operating condition. Janus or directionally sweat-permeable electrodes are currently the most promising for exercise and heavy sweating because they preserve SNR and limit baseline drift, whereas silk-based wet-adhesive electrodes provide the strongest evidence for multi-day ECG/EMG wear. Large-area multichannel arrays are most effective when spatial muscle mapping is required, but their advantage decreases as channel count increases crosstalk, remounting sensitivity and integration complexity. Scalability is strongest for textile, wet-spun and printed electrode architectures; ultrathin cleanroom nanomeshes offer superior conformability but weaker manufacturing scalability. The principal failure modes are sweat-induced delamination and impedance drift, gel dehydration, motion artifacts and loss of skin adhesion. The key trade-off is therefore not permeability alone, but simultaneous retention of breathability, adhesion, low impedance and signal fidelity during sweat and movement.

### 4.3. Biosensors

To fulfill the critical requirements of metabolic monitoring and wound care as outlined in Table 5, breathable bioelectronics extend porous and fibrous electronic platforms from passive heat-moisture regulation to active biochemical interrogation. Unlike dense elastomeric devices, interconnected pores provide continuous pathways for air, water vapor, sweat, wound exudate, and dissolved molecules. Consequently, breathability in biosensors is not merely a comfort-related property; it governs analyte access, sample renewal, and the stability of the local sensing microenvironment.

#### 4.3.1. Non-Invasive Epidermal Biosensing

**Figure 8:**
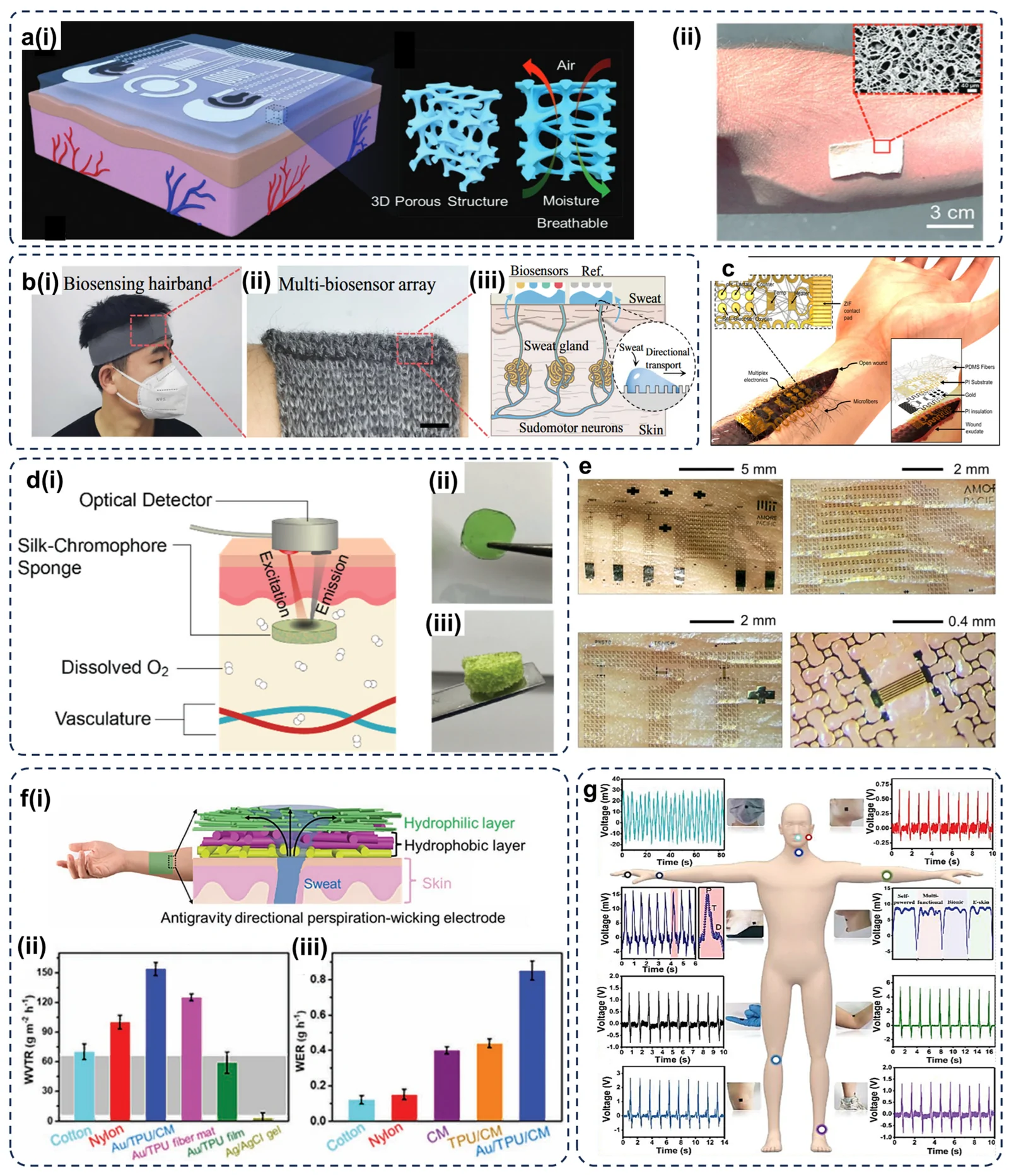
Breathable biosensors and e-skins. (**a**) The FGA-based E-skin: (**i**) A schematic diagram of the e-skin can adhere to the skin surface and the 3D porous network structure, (**ii**) Camera image of the FGA attached to human skin. The inset shows the porous structures of the FGA. Adapted from Ref. [14]. Copyright 2023, WILEY. (**b**) The multi-biosensing hairband system: (**i**,**ii**) The multi-biosensor array within the hairband system. Scale bar, 0.5 cm, (**iii**) Mechanism of the multi-biosensing hairband for directional sweat capture. Adapted from Ref. [149]. Available under a CC-BY 4.0 license. (**c**) e-ECM for inflammation-free chronic wound monitoring. Adapted from Ref. [152]. Copyright 2022, American Chemical Society. (**d**) Phosphorescent oxygen sensors: (**i**) Schematic of a silk-chromophore sponge implanted into subcutaneous tissue to continuously monitor tissue oxygenation, (**ii**) Pictures of silk + PdBMAP film and (**iii**) silk + PdBMAP sponge. Adapted from Ref. [154]. Copyright 2023, Elsevier. (**e**) Optical images of perforated e-skins showing conformal contact on the forearm. Adapted from Ref. [113]. Copyright 2021, American Association for the Advancement of Science. (**f**) The Au/TPU/CM electrode: (**i**) Schematic illustration of design, (**ii**) WVTR and (**iii**) water evaporation rate of films. Adapted from Ref. [158]. Copyright 2022, WILEY. (**g**) Signals of whole-body monitored using the e-skin attached to different parts, which are marked by circles in different colors in the human body model. Adapted from Ref. [160]. Available under a CC-BY 4.0 license.

Non-porous sweat sensors often trap sweat, promote maceration and distort analyte concentration. Breathable architectures mitigate microenvironmental perturbations by combining rapid fluid sampling with continuous vapor dissipation. Zhu et al. developed a flexible GelMA-aerogel (FGA) e-skin whose interconnected capillary network supported heat-moisture exchange and simultaneous monitoring of glucose, lactate, alcohol, hydration, temperature, and biopotentials (Figure 8a) [14]. This work demonstrated that a porous substrate could function as both a physiological interface and a transport medium, rather than serving only as a mechanically compliant support.

Subsequent textile systems shifted the emphasis from passive permeability to controlled sample delivery. Li et al. reported a coaxially wet-spun biosensing hairband (Figure 8b) for pH, Na^+^, K^+^, Ca^2+^, and temperature monitoring, in which the porous sensing yarns promoted directional sweat transport and maintained stable operation for 24 h [149]. Ou et al. further introduced a hierarchical Janus fabric that combined multiplex detection of ions and metabolites with directional perspiration removal and radiative cooling [150]. Its air permeability reached 13 mm s^−1^ and its water-vapor transmission rate reached 468.9 g m^−2^ h^−1^, while the sensing outputs remained stable after repeated bending, shaking, and rinsing. These systems show a transition from breathable substrates to actively regulated sensing microenvironments. However, rapid evaporation may concentrate analytes, and variable sweat rate may alter the effective concentration; compensation for sweat rate, humidity and temperature is therefore required to separate physiological variation from transport artifacts.

Breathable sensing is also expanding beyond liquid biofluids. Shin et al. developed a non-contact epidermal flux sensor that periodically enclosed a small chamber above the skin to quantify outward and inward fluxes of water vapor, volatile organic compounds, and CO_2_ [151]. This approach establishes gas-phase molecular transport as a major, non-invasive diagnostic window, highlighting that open-pore permeability can be dynamically controlled to capture gaseous metabolic exchange without occluding the skin.

#### 4.3.2. Breathable Wound-Interfaced Biosensors

Dynamic chronic wound monitoring demands continuous bio-interfacing with viscous exudate without impairing tissue oxygenation, trapping excess moisture, or damaging newly formed granulation tissue during dressing removal. Brown et al. addressed this challenge with an adhesive-free, open-mesh electronic extracellular-matrix (e-ECM) platform (Figure 8c) that monitored pH, oxygen, glucose, lactate, and temperature while permitting passive gas and fluid transport [152]. Importantly, its response in wound exudate differed from that in buffered solutions because viscosity, oxygen availability, pH variation and biofouling changed the analytical environment. Permeability alone therefore does not guarantee accuracy; calibration must reflect the evolving wound microenvironment.

The field has since advanced from indirect physicochemical indicators toward molecular markers that more directly describe inflammation and tissue repair. Zhao et al. integrated a self-confined tetrahedral-DNA circuit into a porous PAN/TPU nanofiber patch for simultaneous detection of TNF-α, IL-6, TGF-β1, and VEGF [153]. Spatially confined amplification lowered the detection limits by approximately one order of magnitude, while the platform retained stable signals over four weeks. Crucially, this architecture established a spatial decoupling strategy: localized hydrophilic micro-domains sequestered target proteins and supported enzymatic hybridization, while surrounding open nanofibrous networks sustained passive cutaneous respiration over four-week chronic wound trials.

#### 4.3.3. Implantable Permeable Biosensors

For implantable bioelectronics, traditional surface breathability translates into efficient interstitial fluid and gas diffusion, vascularized tissue integration, and bioresorption without eliciting foreign-body capsule formation. Presley et al. embedded an oxygen-sensitive phosphorescent dye into bioresorbable silk films and interconnected porous sponges (Figure 8d) [154]. Although both formats detected physiological oxygen levels during a 28-day implantation study, the porous sponges integrated more effectively with surrounding tissue and responded faster and more consistently to oxygen challenges than dense films. This comparison shows that engineered porosity can reduce diffusional barriers and make the measured signal more representative of the native interstitial environment.

Overall, breathable biosensors are evolving from passive permeable supports into active microfluidic and molecular-transport platforms [155]. Future systems must reconcile pore interconnectivity with stable biorecognition, control evaporation-induced baseline drift and compensate for temperature, pH and sweat-flow cross-sensitivity. Comparisons should therefore be based on performance in the intended biofluid, not buffer-only sensitivity.

### 4.4. Electronic Skins

Breathable e-skins seek to reproduce not only the sensory functions of natural skin but also its exchange of heat, moisture, and gases with the environment. Dense elastomers provide stretchability and intimate contact, yet they may occlude sweat pores, alter local temperature and hydration, and eventually cause signal drift, delamination, or irritation [156]. The development of breathable e-skins has therefore progressed from passive pore networks to directional sweat and heat transport, and finally to multimodal, self-powered, and environmentally compatible systems.

#### 4.4.1. Permeable and Conformal E-Skin Architectures

Achieving intimate epidermal conformability while maintaining low areal mass density requires strategic engineering of structural porosity. Geometrical porosity allows dense functional layers to maintain gas transport while lowering effective flexural rigidity. Ho et al. developed a sugar-templated 3D open-cell scaffold that could be customized from body-surface scans and coated with conductive carbon nanotubes [157]. The porous elastomer had a density of approximately 0.25 g cm^−3^ and supported strain sensing together with EMG, electrodermal activity, and EEG recording. Porosity improved performance indirectly by enabling patient-specific conformity rather than by simply providing air passages. However, interconnected pores alone do not ensure efficient liquid-sweat removal when functional layers or adhesives obstruct transport pathways, and the trade-off between void fraction and electrical/mechanical integrity remains unresolved.

Differentiating gaseous permeability from liquid sweat evacuation, Yeon et al. addressed this limitation by patterning auxetic dumbbell-shaped through-holes across the sensor, interconnect, encapsulation, and adhesive layers [113]. The fully perforated e-skin (Figure 8e) retained approximately 95% of normal transepidermal water loss and allowed liquid sweat to escape directly, whereas partially perforated devices still trapped sweat despite being vapor permeable. The kirigami geometry also reduced the areal mass density to about 1.45 mg cm^−2^ and survived more than 10^6^ deformation cycles, enabling temperature, hydration, strain, and ultraviolet monitoring for over one week without evident irritation. This design demonstrates that vapor permeability cannot substitute for true liquid-sweat permeability. Its main limitation is that extensive perforation reduces active area and complicates high-density circuit layout.

#### 4.4.2. From Passive Breathability to Active Thermal-Moisture Management

Under heavy perspiration or elevated ambient temperatures, passive porous channels can become overwhelmed by excess fluid. Next-generation e-skins incorporate active perspiration wicking and passive radiative cooling to stabilize the sensing interface. Xu et al. introduced an Au/TPU/cellulose membrane with gradients in pore size and surface energy, enabling sweat to be actively transported away from the skin–device interface (Figure 8f(i)) [158]. Its water-vapor transmission (Figure 8f(ii)) and evaporation (Figure 8f(iii)) rates were 2.2 and 7.1 times those of cotton, respectively, while the electrical resistance remained nearly unchanged after 10,000 bending cycles. The improvement reduced hydration and temperature measurement errors, suppressed noise and prevented short circuits in multimodal arrays. This represents a shift from passive permeability to active regulation of the sensing microenvironment, but heat and moisture management must be evaluated for cross-talk with sensing signals rather than treated as isolated comfort functions.

Zhu et al. further demonstrated that porous e-skin matrices can participate directly in microclimate thermal regulation [114]. By leveraging the high solar reflectance and infrared emissivity of 3D gelatin aerogel networks, the breathable e-skin achieved passive radiative cooling, maintaining skin temperatures approximately 5 °C lower than uncovered skin under direct solar exposure while continuously logging physical, electrophysiological, and biochemical signals.

#### 4.4.3. Self-Powered, Multifunctional, and Sustainable E-Skins

Integrating sensing arrays with onboard energy harvesting and end-of-life biodegradability addresses the power supply and e-waste challenges inherent to continuous physiological telemetry. Peng et al. constructed an all-nanofiber triboelectric e-skin by embedding an AgNWs electrode between Poly(lactic-co-glycolic acid) (PLGA) and PVA fiber layers [159]. The hierarchical pores increased the effective contact area for triboelectric sensing while providing channels for thermal-moisture exchange. With an air permeability of approximately 120 mm s^−1^, the device enabled self-powered monitoring of pulse, respiration, vocalization, facial expression, and joint motion, together with antibacterial activity and a tunable degradation lifetime. In this case, porosity served both the electronic function of enhancing contact electrification and the physiological function of maintaining a breathable skin interface.

Further advancing multi-parameter integration within single pixel architectures, Yue et al. developed a spider-web and ant-tentacle doubly bioinspired e-skin [160]. The hierarchical nanofiber network enabled decoupled, self-powered sensing of pressure (0.48 V kPa^−1^), relative humidity, and temperature. Meanwhile, this e-skin can be attached to different parts of the human body to enable the monitoring of various body signals (Figure 8g). This approach demonstrates that multi-stimulus pixels can reduce system complexity, but the reported decoupling must be validated under simultaneous mechanical, thermal and moisture loading rather than sequential stimuli.

Overall, breathable e-skins are evolving from perforated sensors into multifunctional artificial interfaces. No single e-skin architecture is universally superior. Failure in breathable e-skins is most often caused by pore blockage, thermal-moisture fatigue, interfacial delamination, and premature degradation of functional layers. Passive porosity is sufficient for vapor exchange, through-hole or kirigami structures are required for liquid sweat evacuation, and active wicking or radiative cooling becomes necessary under high sweat and heat loads.

Table 5 highlights three application-level conclusions. First, the best-performing material is not necessarily the most deployable: porous hierarchical structures maximize sensitivity, whereas textile and kirigami architectures are generally more scalable and mechanically robust. Second, the most reliable route depends on the dominant stressor: strain-isolated textiles are preferable for daily motion and laundering, Janus electrodes for sweating, active wicking fabrics for exercise biochemistry, open-mesh platforms for wounds, and porous resorbable matrices for implantation. Third, the principal failure modes are strategy-specific: unstable contacts and microcracks in random mechanical networks, sweat-induced interfacial degradation in electrophysiological sensors, flow- and biofouling-dependent calibration in biosensors, and pore blockage or premature degradation in multifunctional e-skins. These differences, rather than a single sensitivity ranking, should guide material and structural selection.

Across the selected cases, durability is mostly demonstrated by one stressor at a time: 1000–40,000 mechanical cycles, 1–7 days of wear, 4–10 washing cycles, or 28 days of implantation. No system has yet been evaluated after combined sweat exposure, washing, mechanical wear and aging, so the available results establish prototype robustness rather than service-life reliability.

**Table 5:** Benchmarking Breathable Electronics: From Technical Targets to Healthcare Applications.

Primary Life & Health Area	Sensing Modality & Target	Design Strategy	Key Sensing Performance	Reliability Evidence	Main Limitation	Refs.
Respiratory & Tactile Perception	Dynamic contact pressure	PU/Au/parylene nanomesh capacitor	Sensitivity: 0.141 kPa^−1^ (<1 kPa)	<0.15% change after 1000 cycles at 19.6 kPa	Sensitivity decreases to 0.010 kPa^−1^ at >10 kPa	[36]
Full-body Motion & Rehabilitation	Large-range joint strain	Deterministic-contact braided textile	GF 140, sensing range 50%	40,000 cycles at 50% strain; washable	Sensitivity decreases at high strain	[37]
Cardiovascular & Neuromuscular Health	Long-term dry ECG/EMG	Glycerol-plasticized silk/PEDOT:PSS	Stable ECG/EMG; stretchability >250%	WVTR 117 g m^−2^ h^−1^ at 37 °C; durability >1 week	Repeated-wash and long-term aging data remain limited	[140]
Neurological & Epidermal Monitoring	Motion/Sweat-tolerant EMG	15 um Janus HPAN/PU/AgNW electrode	EMG SNR loss during sweating only 0.35 dB	Sweat transport <5 s; 4200 bending cycles, ΔR ≈ 1.5%	Multiday wear and scalable Janus fabrication are not demonstrated	[141]
Metabolic & Sweat Biomarkers	Multi-ion/metabolite analysis	Hierarchical Janus woven fabric	Multiplex sensing of Na^+^, K^+^, pH, lactate, uric acid	WVTR 468.9 g m^−2^ h^−1^; air permeability 13 mm s^−1^; stable over 7 days	Output remains sensitive to sweat flow and temperature	[150]
Internal Tissue & Post-operative Care	Implantable deep-tissue O_2_	Bioresorbable silk film versus porous sponge	KSV = 2.7–3.2 × 10^4^ M^−1^	28-day rodent implantation;	Degradation control and immune response remain unresolved	[154]

## 5. Integration Strategies for Breathable Electronic Systems

### 5.1. Integration Strategies

#### 5.1.1. Circuit Design and Signal Processing

Circuit integration in breathable electronics involves more than signal amplification and digitization; it requires a balance among signal fidelity, computational capability, mechanical compliance, power consumption, and preservation of effective breathable area. A complete signal chain generally consists of sensor readout, analog front-end conditioning, analog-to-digital conversion, digital processing, and data storage. However, each additional circuit module introduces conductive traces, active devices, interconnects, and encapsulation layers that may locally increase stiffness and obstruct air- and water-vapor transport. Therefore, circuit architectures for breathable electronics should be evaluated not only by electrical performance, but also by how effectively they localize rigid components, minimize material coverage, and maintain porous or fibrous transport pathways.

The analog front end must be matched to the transduction mechanism of the sensor. Electrophysiological electrodes require low-noise, high-input-impedance amplification, electrochemical sensors generally require current-to-voltage conversion, and resistive or capacitive sensors require corresponding readout interfaces. After conditioning, the signals are digitized for calibration, feature extraction, storage, and communication. Thus, the integration challenge is not simply to miniaturize individual circuits, but to accommodate heterogeneous signal types within a low-power and mechanically compliant architecture.

One strategy is to replace conventional rigid silicon circuitry with intrinsically flexible active electronics. Conventional analog front ends rely primarily on silicon-based integrated circuits. Although these circuits offer high performance and mature manufacturing processes, rigid chips and their supporting circuitry cannot fully accommodate the bending, twisting, and stretching of skin and textiles. To improve mechanical compliance, organic semiconductors, metal oxides, carbon nanotubes, graphene, and 2D transition-metal dichalcogenides are used to construct flexible thin-film transistors and, subsequently, amplifiers, filters, and analog-to-digital conversion circuits [161]. Organic semiconductors are compatible with low-temperature and printing processes; metal oxides provide relatively high carrier mobility; and carbon nanotubes and 2D materials combine favorable electrical properties with mechanical flexibility. The principal advantage of this route is mechanical homogeneity: active devices and signal-conditioning circuits can deform together with the underlying substrate, thereby reducing the stiffness mismatch associated with conventional chips. However, this advantage is currently offset by lower device uniformity, limited circuit complexity, environmental sensitivity, and lower manufacturing yield than mature silicon electronics. These limitations become increasingly important as circuit complexity rises from simple amplifiers toward high-resolution analog-to-digital conversion, memory, and digital computation.

**Figure 9:**
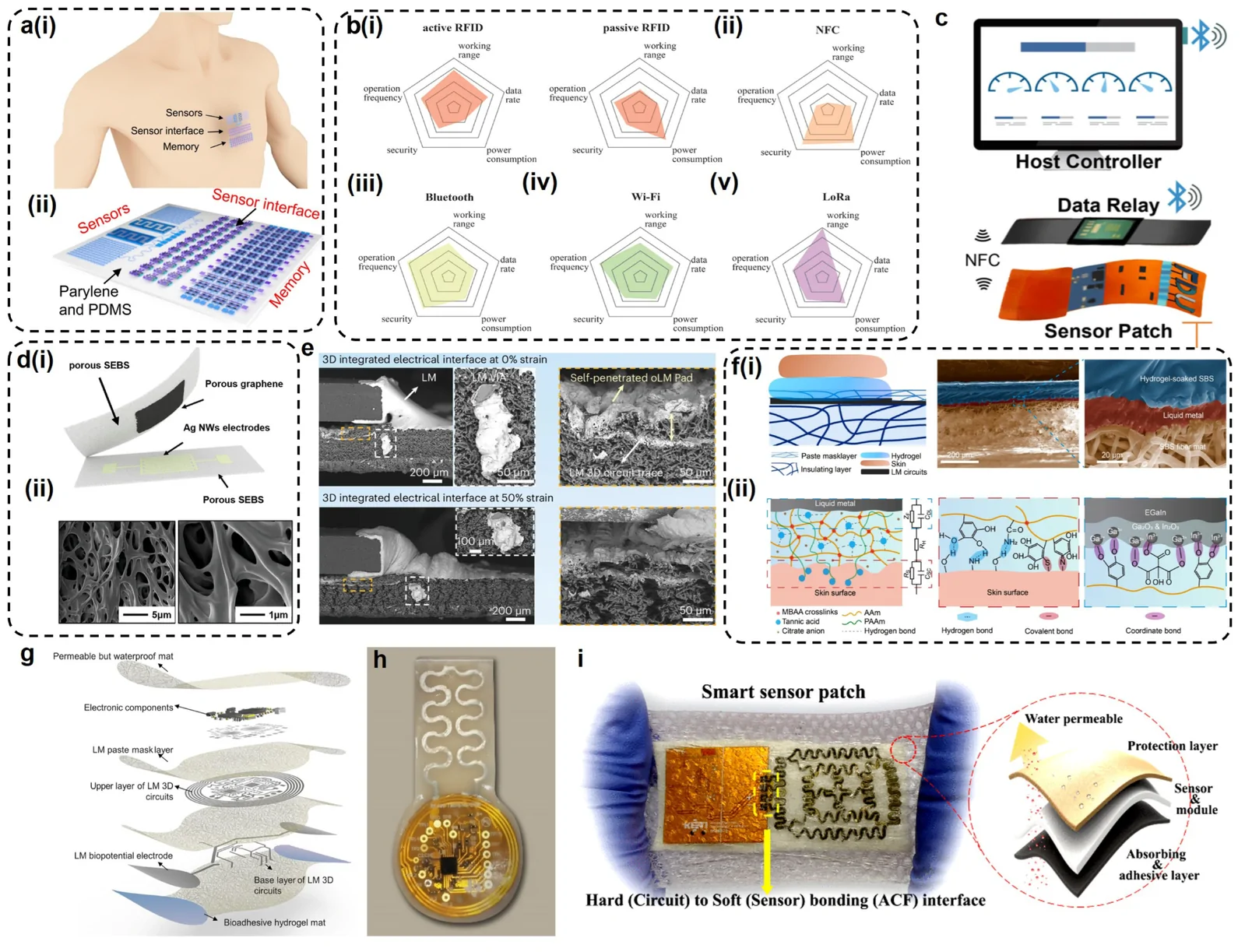
Representative integration strategies and system configurations of breathable electronics. (**a**) Epidermal electronic system: (**i**) skin-mounted system integrating flexible sensors, interface circuits, and memory, (**ii**) spatial layout of sensors, interface circuits, and the flexible flash-memory array. Adapted from Ref. [162]. Copyright 2022, American Association for the Advancement of Science. (**b**) Comparison of representative wireless technologies: (**i**) RFID, (**ii**) NFC, (**iii**) Bluetooth, (**iv**) Wi-Fi, and (**v**) LoRa. Adapted from Ref. [165]. Copyright 2024, WILEY. (**c**) Hybrid wireless system combining NFC powering/readout with BLE relaying. Adapted from Ref. [163]. Copyright 2023, MDPI. (**d**) Porous SEBS-based breathable encapsulation: (**i**) wearable device encapsulated with porous SEBS, (**ii**) hierarchical pore structure of SEBS. Adapted from Ref. [168]. Available under a CC-BY 4.0 license. (**e**) Soft–hard electromechanical interface based on LM interconnects. Adapted from Ref. [169]. Copyright 2024, Springer Nature. (**f**) Hydrogel interface: (**i**) multilayer skin–hydrogel–LM interface, (**ii**) interfacial interactions between the hydrogel, skin, and LM. Adapted from Ref. [39]. Copyright 2024, American Association for the Advancement of Science. (**g**) Layered architecture of the breathable cardiac e-skin BreaCARES. Adapted from Ref. [170]. Copyright 2025, American Association for the Advancement of Science. (**h**) Battery-free Ni-GaIn/NFC multisensing skin patch. Adapted from Ref. [40]. Available under a CC-BY 4.0 license. (**i**) Soft–hard integrated smart patch based on a kirigami-serpentine architecture. Adapted from Ref. [41]. Available under a CC-BY 4.0 license.

Carbon-nanotube thin-film transistors provide a representative route toward integrating flexible signal processing and data storage. Xiang et al. demonstrated this route by integrating humidity, temperature, and ECG sensors with a carbon-nanotube thin-film-transistor differential amplifier and flexible flash memory on a single epidermal platform (Figure 9a) [162]. The amplifier achieved a voltage gain of approximately 27 dB and a common-mode rejection ratio above 43 dB, while the memory exhibited an estimated endurance exceeding 10^6^ cycles. This system illustrates the feasibility of combining sensing, amplification, and local storage within a mechanically flexible platform. Importantly, placing the front-end electronics close to the sensors also shortens weak analog-signal paths and can reduce noise introduced by long interconnects.

However, mechanical flexibility should not be equated with breathability. The primary contribution of this system is the integration of flexible sensing, amplification, and storage rather than preservation of an open mass-transport pathway. Its functional units are supported by approximately 2-μm Parylene and 10-μm continuous PDMS films, which improve softness and conformability but may still restrict water-vapor transport. Therefore, such flexible active circuitry should be regarded as a functional building block rather than a complete solution for breathable electronics. To translate this concept into a breathable system, active circuitry should be combined with porous or fibrous substrates, mesh-patterned interconnects, reduced circuit coverage, or localized encapsulation.

After analog-to-digital conversion, the digital circuitry also performs channel control, data readout, preliminary processing, and power management. In the system developed by Feng et al., the flexible sensing patch integrates sensor interfaces, an analog-to-digital converter, and a digital control unit [163]. The digital control unit switches among IDLE, READY, and ACTIVE states and disables analog-to-digital conversion and most communication circuitry during idle periods, thereby reducing unnecessary energy consumption. These results indicate that low-power design for breathable electronics must consider not only the static power consumption of individual components, but also the operating duration of the entire signal chain, which can be reduced through on-demand sampling, sleep–wake control, and local data processing.

A second and currently more practical strategy is rigid-flex heterogeneous integration, in which mechanically compliant sensing and interconnection layers are combined with localized conventional silicon electronics. This approach is particularly suitable for functions that still rely on mature silicon technology, such as high-quality signal conditioning, analog-to-digital conversion, memory, wireless control, and complex computation. In textile hybrid electronics, flexible sensors, conductive fibers, and large-area interconnects are distributed throughout a breathable textile, while the rigid chips required for signal conditioning, computation, and storage are concentrated in a small number of localized regions. This arrangement assigns sensing and interconnection to the soft substrate and processing and control to the rigid chips [164]. Such a layout exploits the performance of mature silicon devices while shortening weak analog-signal paths and avoiding large-area coverage by conventional rigid circuit boards.

However, this performance advantage is accompanied by a system-level trade-off. Each rigid island, interposer, solder joint, and locally encapsulated region reduces the effective open area of the breathable substrate and introduces a mechanical discontinuity at the soft–hard interface. Increasing the number or size of rigid modules may improve computational and communication capability, but simultaneously increases local stiffness, stress concentration, and obstruction of air- and water-vapor transport. Therefore, the key design objective is not to eliminate rigid electronics completely, but to minimize and strategically localize them while preserving sufficient breathable pathways around the active regions.

Soft–hard interconnection represents a major reliability bottleneck in rigid-flex breathable systems. The primary challenge is to maintain low electrical resistance while avoiding excessive local stiffness, stress concentration, and blockage of porous transport pathways. Continuous interposers provide relatively high wiring density, but locally suppress substrate porosity; dividing the circuit into smaller interposer modules improves mechanical compliance, although it increases the number of interfaces and the total occupied area. Conventional soldering offers low contact resistance, but the resulting rigid joints are susceptible to stress concentration and fatigue under repeated bending and stretching. In contrast, LM-based interconnections better accommodate deformation and reduce mechanical mismatch between rigid chips and compliant conductors, although additional fixation may still be required to prevent chip displacement. Therefore, soft–hard interconnections should be evaluated not only by their initial electrical conductivity, but also by occupied area, local stiffness, fatigue resistance, and their influence on system-level breathability.

Overall, two complementary circuit-integration routes are emerging for breathable electronics. Fully flexible active circuitry minimizes mechanical mismatch and is attractive for low-complexity functions distributed over deformable surfaces, but its wider adoption is still limited by device variability, environmental stability, circuit complexity, and manufacturing yield. Rigid-flex heterogeneous integration retains the performance and manufacturing maturity of silicon electronics and is currently more suitable for functions requiring high-quality amplification, analog-to-digital conversion, memory, and computation; however, rigid islands and their interconnections inevitably reduce local breathable area and introduce mechanical discontinuities. Consequently, the two approaches should not be viewed as mutually exclusive. A practical system-level strategy is to distribute sensors and compliant interconnects over porous or fibrous regions while localizing high-performance rigid electronics in small, low-strain areas. Together with low-power operation, mesh-patterned interconnects, and reliable soft–hard interfaces, this architecture provides a route to balancing signal quality, computational capability, effective porosity, and long-term wearing comfort.

#### 5.1.2. Integration of Wireless Communication and Power Supply

Wireless communication and power supply must be co-designed in breathable electronic systems because communication range, data throughput, and operating duration directly determine energy demand, while batteries, antennas, coils, and wireless chips occupy area and may increase local stiffness or obstruct vapor-transport pathways. Therefore, wireless technologies should be selected according to data volume, transmission distance, power budget, and compatibility with porous or fibrous substrates rather than communication performance alone. In addition, antenna layout, body-induced radio-frequency effects, and skin deformation must be considered because they can simultaneously affect mechanical conformity and wireless-link stability [165].

Wireless communication technologies commonly used in flexible wearable systems include Bluetooth Low Energy (BLE), radio-frequency identification (RFID), NFC, Wi-Fi, and Long Range (LoRa), each of which offers distinct characteristics in communication range, data rate, power consumption, and system volume. As summarized in Table 6, NFC and RFID are attractive for thin, battery-free patches but are limited by short communication range and alignment sensitivity. BLE offers a practical compromise between continuous smartphone connectivity and moderate power demand, whereas Wi-Fi supports higher data throughput at the cost of greater energy consumption and thermal load. LoRa extends communication range with low data throughput, and is therefore better suited to intermittent remote monitoring. These differences indicate that no single protocol is universally optimal; the appropriate choice depends on sensing bandwidth, monitoring distance, and available energy (Figure 9b). Instead, protocol selection should be matched to communication distance, data requirements, available energy, and system-level integration constraints [165]. Feng et al. further demonstrated an NFC and BLE relay architecture in which the skin-mounted sensing patch remains battery-free while BLE is used for longer-range data transmission, illustrating how hybrid wireless strategies can combine the advantages of different protocols [163]. These comparisons indicate that wireless design in breathable patches should be evaluated together with antenna layout, power supply, mechanical compliance, and overall system breathability rather than as an isolated communication choice.

**Table 6:** Comparison of Wireless Communication Technologies.

Technology	Range	Data Rate	Power Demand	Battery-Free	Power Supply	Breathability Compatibility	Advantages	Limitations	Applications	Refs.
NFC	Short	Low–moderate	Very low-low	Yes	RF harvesting/reader	High	Battery-free; compact	Short range; alignment sensitive	Epidermal patches; intermittent sensing	[163,165,166]
RFID	Short-moderate	Low	Very low	Yes	RF harvesting/reader	High	Low power; simple	Low data rate; antenna dependent	Identification; low-rate sensing	[165,167]
BLE	Moderate	Moderate	Low–moderate	Generally no	Battery/storage	Moderate	Smartphone compatible; continuous link	Requires storage; local rigidity	ECG/EMG; motion; multiparameter monitoring	[163,165]
Wi-Fi	Moderate	High	High	No	Battery/storage	Low–moderate	High throughput	High power; thermal load	High-bandwidth sensing; imaging	[165]
LoRa	Long	Low	Low	Generally no	Battery/storage	Moderate	Long range; low power	Low data rate; larger antenna	Remote and intermittent monitoring	

Wireless modules must also be structurally compatible with breathable substrates. Hybrid integration connects flexible sensors and antennas with conventional wireless chips, providing mature communication performance, but introducing localized rigid components and additional interfaces. Monolithic integration reduces external circuitry by combining sensing and wireless functions within the same structure, but requires stricter material and fabrication control [167]. In either case, mesh-patterned, fibrous, or locally patterned antennas are preferable to large-area dense conductors because they preserve vapor-transport pathways. However, increasing antenna porosity can reduce conductive area and alter impedance matching, while deformation, sweat, and human tissue may shift resonance and attenuate the wireless signal. Thus, antenna breathability and communication reliability must be optimized together.

Feng et al. [163] demonstrated this communication-power co-design using a three-tier architecture comprising a battery-free sensing patch, a wireless relay, and a host unit. The relay powers and reads the patch through NFC and then forwards the data through BLE (Figure 9c). By relocating the battery and Bluetooth module away from the skin-mounted patch, this architecture reduces patch thickness and stiffness while overcoming the short communication range of NFC. Its main limitation is the continued dependence on a nearby relay and stable NFC coupling.

Because communication capability is constrained by available energy, wireless and power architectures should be selected together. Battery-based systems provide stable power for continuous BLE or high-data-rate communication but increase thickness, weight, stiffness, and surface coverage. By contrast, RF/NFC power transfer can support thin battery-free patches, although the available power and operating distance are limited by coupling efficiency and alignment. Optical and ultrasonic power transfer provide alternatives for specialized surface-mounted or implanted systems, but their use depends strongly on illumination, tissue depth, and coupling conditions [166].

Energy harvesting from body motion or heat can further improve autonomy, but piezoelectric, triboelectric, and thermoelectric outputs are strongly dependent on activity level or temperature gradients and are generally insufficient for continuously powering high-consumption communication modules. These approaches are therefore more suitable for low-power sensing or for replenishing a storage element than for completely replacing batteries. At the system level, the practical goal is to match communication duty cycle and power-management strategy to the available energy rather than maximizing either communication range or harvested power independently.

Overall, wireless communication and power supply in breathable electronics should be treated as a coupled system-design problem. High-bandwidth or continuous communication improves data accessibility but increases energy-storage and thermal-management requirements, whereas battery-free short-range schemes reduce device burden at the expense of communication distance and available power. Future integration should therefore prioritize low-power operation, porous or patterned antennas, localized rigid modules, and communication-energy matching to preserve system-level breathability during long-term wear.

#### 5.1.3. Encapsulation and Interface Technologies

Encapsulation and interface design in breathable electronics involves an inherent trade-off between environmental protection and physiological permeability. Encapsulation must protect sensors, interconnects, and electronic components from sweat, moisture, oxidation, and mechanical damage, while the skin interface must maintain stable adhesion and low electrical impedance during motion and perspiration. However, continuous dense encapsulation or adhesive layers can obstruct air- and water-vapor transport, whereas excessive porosity may weaken electrical insulation, moisture resistance, and interfacial contact. Therefore, breathable systems require selective protection and interface engineering, rather than conventional large-area sealing.

Representative encapsulation and interface materials provide different combinations of protection, flexibility, and permeability [168]. Dense elastomers such as PDMS and Ecoflex offer electrical insulation and moisture protection but restrict vapor transport when used over large areas. Parylene provides an effective thin barrier but has limited intrinsic stretchability. Porous SEBS can better preserve breathability while maintaining mechanical compliance, whereas hydrogels provide soft adhesion and ionic contact but are susceptible to dehydration, swelling, and mechanical degradation. These differences suggest a functional division of materials: dense barriers are better suited to localized protection of chips and joints, porous elastomers to breathable coverage, and hydrogels to skin-contact interfaces.

At the skin–device interface, adhesion, breathability, and electromechanical stability must be optimized simultaneously [17]. Strong attachment suppresses relative motion and fluctuations in contact impedance, but continuous adhesive layers may block pores and promote sweat accumulation. Conversely, excessive open area can reduce effective contact and weaken signal stability. Porous fibrous substrates, nanomesh electrodes, and hydrogel interfaces help maintain vapor transport while preserving electrical contact, whereas serpentine interconnects, LMs, and conductive networks redistribute strain and reduce delamination or interconnect failure. Thus, interface design must balance contact area, permeability, and deformation tolerance rather than maximizing any single parameter.

Zhuang et al. demonstrated that high-density integration can remain compatible with breathability by combining a fibrous substrate, multilayer LM interconnects, vertical connections, and hybrid LM solder [169]. This architecture integrates rigid electronic components with compliant circuitry while preserving open regions for vapor transport (Figure 9e). The study illustrates that multilayer integration does not necessarily eliminate breathability, provided that rigid components and interconnect coverage are spatially localized and mechanically decoupled from highly deformable regions.

Yao et al. further demonstrated coordinated encapsulation and interface design using an electrospun SBS fibrous mat, LM circuits, and a conductive bioadhesive hydrogel [39]. The porous fibrous layer provides breathable mechanical protection, the LM accommodates deformation, and the hydrogel maintains adhesive electrical contact with the skin (Figure 9f). Compared with continuous dense encapsulation, this combination preserves vapor pathways across the substrate, conductor, and interface layers, thereby reducing sweat accumulation and contact-impedance drift.

Overall, encapsulation in breathable electronics should shift from large-area continuous sealing toward localized, selective, and porous protection. Vulnerable components, such as chips, joints, and interconnects require targeted encapsulation, whereas major skin-contact regions should preserve fibrous, porous, or mesh-like pathways for air and vapor transport. At the same time, soft adhesive interfaces and strain-tolerant interconnections are required to maintain low impedance and mechanical reliability. Accordingly, system-level evaluation should consider not only air permeability and water-vapor transmission, but also adhesion strength, interfacial impedance, cyclic electrical stability, sweat or wash resistance, and long-term skin compatibility.

### 5.2. Major Application Formats

#### 5.2.1. Textile-Based Integration

Textile-based integration exploits the intrinsic porosity, flexibility, and large-area deformability of fibrous substrates to host sensing, interconnection, processing, communication, and power modules while preserving air- and water-vapor transport. Unlike dense elastomeric platforms, textiles provide distributed open pathways without requiring extensive perforation. At the system level, two complementary integration routes can be distinguished: all-fiber/all-textile architectures that prioritize breathability and mechanical compliance, and rigid-flex heterogeneous architectures that prioritize functional density and electronic performance.

In rigid-flex textile systems, large-area sensing and interconnection are assigned to soft fibrous structures, whereas signal conditioning, computation, storage, and wireless communication are localized in conventional microelectronic components. The textile hybrid-electronics framework described by Cai et al. follows this functional partitioning principle, allowing mature silicon electronics to be incorporated without covering the entire textile with rigid circuitry [164]. The key advantage is high system functionality; the corresponding trade-off is the introduction of local stiffness, additional interfaces, and reduced effective breathable area around rigid modules.

Ma et al. demonstrated monolithic textile integration by combining potassium-ion sensing, signal conditioning, a microcontroller, and Bluetooth communication on the same porous textile [38]. The system retained an air permeability of 79 mm s^−1^ and a water-vapor transmission rate of 270 g m^−2^ d^−1^, showing that patterned conductors and localized circuit integration can preserve textile porosity while supporting complete sensing-to-wireless functionality. This approach is attractive for wearable systems requiring continuous data transmission, although the integrated electronic regions still introduce local stiffness and fabrication complexity.

An alternative route is all-fiber integration. Li et al. fabricated a self-powered e-skin by scalable electrospinning, in which the fibrous network simultaneously serves as the supporting, triboelectric, and sensing structure [34]. The device maintained stable operation at 50% deformation and achieved a water-vapor transmission rate of 10.26 kg m^−2^ d^−1^. By avoiding dense films and rigid electronic modules, this architecture offers high breathability and compatibility with continuous large-area fabrication. However, its integrated functions remain mainly limited to sensing and energy conversion, while complex processing, storage, and wireless communication generally require additional electronics.

These two examples reveal the central trade-off in textile integration. All-fiber architectures maximize permeability and manufacturing continuity but offer limited circuit complexity, whereas rigid-flex architectures provide more complete electronic functionality at the cost of localized stiffness and reduced effective breathable area.

Higher functional density has been demonstrated by Yao et al., who integrated 128 independently addressable stimulation pixels, LM multilayer circuits, rigid control electronics, and Bluetooth communication into a breathable fibrous textile [39]. The system achieved approximately 400% stretchability, a water-vapor transmission rate above 657 g m^−2^ d^−1^, and an air permeability above 40 mm s^−1^. This example shows that textile platforms can support not only sensing but also multichannel actuation and wireless human–machine interaction, although such integration requires more complex multilayer fabrication and soft–hard interconnection.

For long-term physiological monitoring, Zhuang et al. developed the BreaCARES cardiac e-skin by integrating a fibrous adhesive interface, multilayer LM circuitry, rigid electronics, power management, and BLE communication [170]. The complete system is approximately 180 μm thick and weighs about 0.489 g, while supporting ECG acquisition, processing, and wireless transmission for nine consecutive days (Figure 9g). Its fibrous architecture preserves substantially higher vapor and air transport than conventional ECG electrodes, demonstrating the potential of textile-derived integration for thin, clinically oriented monitoring systems.

In conclusion, textile-based integration can be divided into two complementary routes. All-fiber or all-textile architectures offer superior breathability, softness, and scalability for large-area sensing or self-powered functions, but currently support limited processing and communication complexity. Rigid-flex or monolithic textile systems provide more complete sensing, computation, wireless communication, and actuation, but introduce localized stiffness, additional interfaces, and partial loss of breathable area. Therefore, the preferred architecture depends on the required functional density. Low-complexity distributed sensing favors all-fiber approaches, whereas multifunctional closed-loop or continuously connected systems benefit from localized rigid electronics. Future development should focus on scalable high-resolution patterning, wash- and fatigue-resistant soft–hard interconnections, and system-level breathability testing after complete electronic integration.

#### 5.2.2. Patch-Based Integration

Patch-based integration concentrates sensors, flexible interconnects, signal-processing circuitry, wireless communication, and power-supply modules within a limited area and attaches the complete system directly to the skin. Compared with textile-based systems, patches conform more closely to skin topography and are suitable for acquiring electrophysiological, temperature, pressure, humidity, and biochemical signals. However, high component coverage and continuous adhesive layers also readily cause sweat accumulation, interfacial delamination, and signal drift. Patch-based systems therefore require not only multifunctional integration, but also porous substrates, island-bridge architectures, serpentine interconnects, or kirigami structures to preserve air- and water-vapor-transport pathways, while rigid chips are concentrated in localized regions [20].

Early patch-based systems primarily combined flexible sensors with separate wireless modules. Lee et al., for example, integrated stretchable epidermal EMG electrodes with a wireless readout module [171]. Although this architecture enabled untethered signal acquisition, its continuous PDMS substrate remained nonporous. This distinction highlights an important transition in patch design: mechanical flexibility and wireless operation alone are insufficient, and later systems increasingly incorporate breathability, reduced battery burden, and long-term interface stability.

Lin et al. use a NiGaIn semiliquid alloy to fabricate a serpentine strain sensor and combine it with an NFC tag that integrates a thermistor, a photoresistor, and interface circuitry, thereby constructing a battery-free multisensor patch (Figure 9h) [40]. The NFC tag harvests energy from an external reader while performing signal processing and wireless transmission, allowing the patch to monitor temperature, illumination, finger bending, and swallowing. This design reduces the thickness and stiffness introduced by batteries and external wires. However, the near-field communication range remains limited, and the coil and chip still occupy a portion of the skin area. Therefore, circuit patterning and localized module layout are required to further reduce their impact on breathability.

For long-term monitoring, Zhang et al. [172] mix graphene oxide with cellulose fibers and use the 3D hierarchical pores of the fibrous network to construct temperature and pressure sensors. The sensors further connect to a flexible printed circuit board, and Bluetooth transmits the data to a smartphone. The device monitors body temperature, pulse, swallowing, coughing, and vocalization and maintains stable function after water immersion and repeated washing-drying cycles. This work demonstrates that a breathable porous substrate simultaneously supports sensing and skin contact. However, the flexible printed circuit board and wireless module remain localized dense regions and are therefore better positioned at the patch edge or away from the primary contact area.

Structural design also mitigates the mechanical mismatch between rigid electronic modules and skin deformation. Lee et al. construct a smart patch based on a kirigami-serpentine heterogeneous architecture that integrates temperature, humidity, and acceleration sensors, signal-processing circuitry, and a Bluetooth Low Energy module within the same system [41]. The kirigami structure accommodates skin stretching through geometric unfolding while providing pathways for sweat release, allowing temperature, humidity, and motion data to be continuously transmitted to a mobile terminal. This scheme does not require every chip to be intrinsically stretchable, but the cut dimensions, sensor positions, and rigid-module layout require coordinated design to avoid local stress concentrations and loss of attachment area (Figure 9i).

Patch platforms are also evolving from passive monitoring toward closed-loop therapeutic systems. Liu et al. integrated microfluidics, organic electrochemical transistor sensors, stimulation electrodes, and flexible control electronics into a wireless wound patch capable of sensing infection-related signals and delivering electrical stimulation [173]. The system integrates physiological sensing, signal-based decision making, and therapeutic intervention within a single platform, demonstrating the high functional density achievable in patch-based systems. However, each additional sensing, actuation, control, and encapsulation layer increases thickness and surface coverage, making preservation of gas exchange particularly challenging in wound-contact regions.

Together, these examples illustrate a progression from simple wireless sensing to battery-free operation, breathable and washable monitoring, mechanically decoupled heterogeneous integration, and finally closed-loop therapeutic functionality. Increasing functional density, however, consistently intensifies the trade-off between system capability and breathable area.

In a word, patch-based integration is particularly suitable for localized physiological monitoring and therapeutic intervention because it provides intimate skin contact and high functional density within a compact area. Its main limitation is that increasing numbers of rigid chips, coils, batteries, adhesives, and encapsulation layers progressively reduce system-level breathability and increase local stiffness. Practical designs should therefore spatially separate dense electronic modules from primary skin-contact regions and use porous sensing layers, open or mesh interconnects, localized rigid islands, and selective encapsulation. Unlike substrate-level characterization alone, evaluation should focus on the complete assembled patch, including breathability, sweat tolerance, adhesion stability, mechanical durability, and long-term signal retention.

As summarized in Table 7, textile- and patch-based systems exhibit distinct integration trade-offs. Textile platforms generally preserve larger breathable areas and provide better compatibility with distributed sensing, large deformation, and large-area fabrication, whereas their circuit density and interconnection complexity remain constrained. Patch-based systems, in contrast, enable more compact integration of sensing, processing, wireless communication, and therapeutic functions, but the increased use of rigid chips, coils, batteries, and encapsulation layers can reduce system-level breathability. Across both formats, higher functional density is therefore commonly accompanied by greater local stiffness, power demand, and fabrication complexity. These comparisons highlight that the optimal integration strategy depends on the required balance among functionality, breathability, mechanical compliance, and long-term reliability.

**Table 7:** Comparison of Different Integration Strategies.

Integration Form		Circuit & Communication	Power Supply	Encapsulation & Interface	Breathability Compatibility	System Complexity	Limitations	Refs.
Textile-based	128-pixel haptic textile	LM/HV driver/BLE	Li-battery/Boost converter	SBS encapsulation/Hydrogel interface/LM solder	Fiber porosity/Breathable	Very High (System)	Complex HV circuit; washability unverified	[39]
	Sweat K^+^ textile wristband	Cu circuit/Amp/Filter/ADC-MCU/BT	3.7 V Li-battery	Local Ecoflex/PDMS microfluidics	Preserved fiber pores/Local encap.	High (System)	Single analyte; long-term washability	[38]
	All-fiber self-powered e-skin	Triboelectric pressure sensing	Triboelectric self-powered	All-fiber layered structure	Electrospun porous network	Low-Mid (Device)	No signal processing/storage/comm	[34]
	Clinical-grade ECG e-skin	3D LM/MAX30003/BLE	Inductive or Li-battery	Hydrogel fiber/LM solder/SBS encap.	Fiber micropores/Breathable	Very High (System)	Single lead limit; battery life issues	[170]
Patch-based	CNT epidermal sensing	CNT sensor/Diff amp/Flexible flash	External power	Parylene/PDMS ultra-thin	Ultra-thin; non-porous	Mid-High (Device)	No ADC/control/wireless; non-breathable	[162]
	Multimodal sweat wireless sys.	FPC + Local PCB/AFE/ADC/FPGA/NFC-BLE	NFC + Relay battery	Flexible patch/Local rigid circuit	LIG porous/Data lacking	High (System)	Relay dependent; distance limit	[163]
	3D breathable E-skin	3D LM/Vertical interconnect/NFC	Battery or NFC	Hybrid LM solder/Fiber substrate	Fiber pores/Low coverage	Very High (System)	Complex fabrication; reusability	[169]
	Wireless EMG system	Epidermal electrode/ADC/MCU/BT	500 mAh battery	PDMS substrate/Medical adhesive	Non-porous	Mid (Module)	Wires present; 5h battery life	[171]
	Battery-free NFC patch	Ni-GaIn/NFC chip/ADC-FRAM	NFC RF power	Silicone substrate/Ecoflex	Serpentine/Non-breathable	Mid-High (Module)	8cm range; contains rigid parts	[40]
	Breathable washable patch	Graphene-Cellulose/FPCB/BT	Unspecified	Porous electronic paper/FPCB	3D hierarchical pores	Mid-High (System)	Local rigidity; power undefined	[172]
	Kirigami multi-parameter patch	Temp/Humid/Accel/FPCB/BLE	Battery	ACF/Layered encap./Adhesive	Kirigami slits/Sweat transport	High (System)	Rigid IC/battery dependent	[41]
	Closed-loop wound patch	Microfluidics/OECT/ADC-BLE/Stim	PMU	FPCB/Silicone elastomer	Non-breathable	Very High (Closed-loop)	Concept only; poor breathability	[173]

## 6. Long-Term Failure Mechanisms and Reliability Enhancement

Although breathable electronics have demonstrated tremendous application potential in continuous health monitoring and bionic e-skins, their long-term stability in complex physiological and environmental conditions remains a significant bottleneck. Transitioning from laboratory prototypes to reliable clinical-grade applications requires a systematic understanding of long-term failure mechanisms and the development of corresponding reliability enhancement strategies.

**Figure 10:**
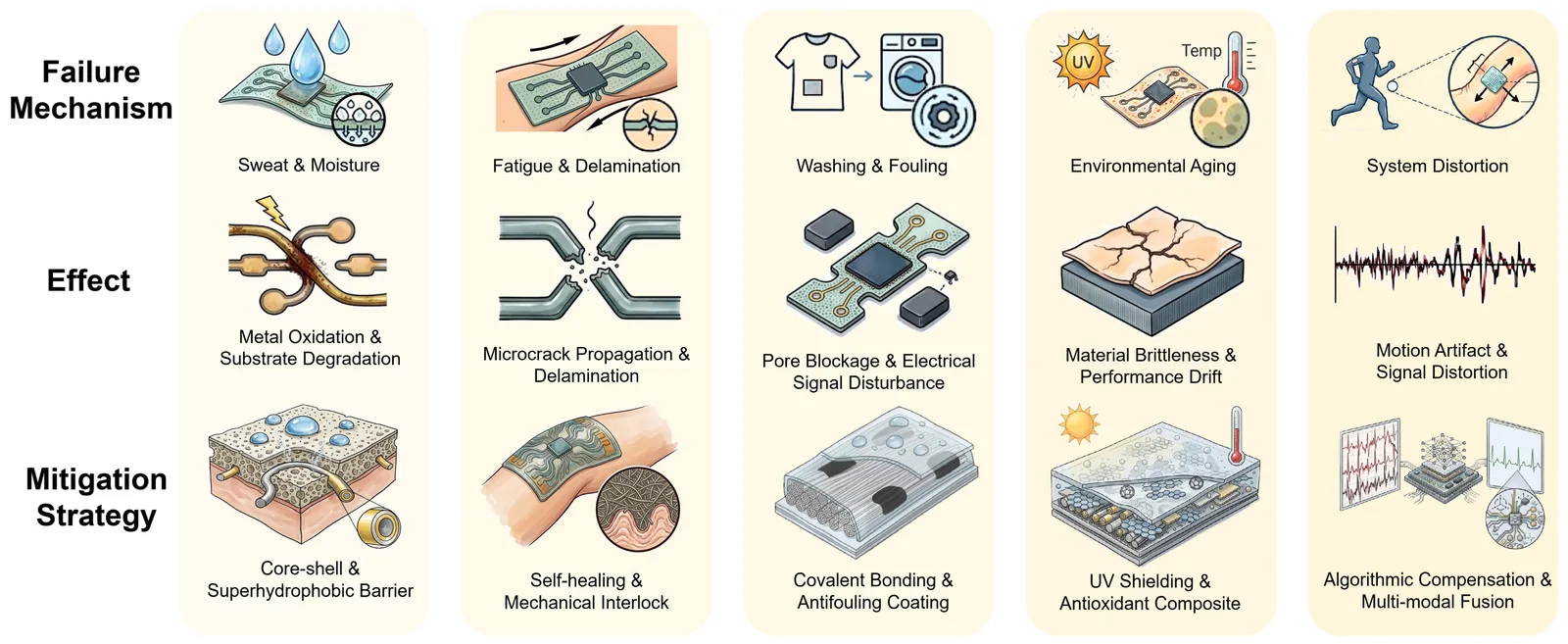
Schematic illustration of multi-scenario failure mechanisms, failure effects, and active mitigation strategies for breathable electronics.

To establish a comprehensive framework for reliability design, Figure 10 systematically maps the primary degradation pathways and corresponding mitigation strategies for breathable epidermal electronics. It categorizes these operational and environmental challenges into five interconnected scenarios: Sweat Corrosion and Moisture Failure (Section 6.1), Mechanical Fatigue, Wear, and Delamination (Section 6.2), Washing Damage and Anti-Fouling Strategies (Section 6.3), Environmental Aging and Weatherability (Section 6.4), and System Distortion and Algorithmic Compensation (Section 6.5). For each scenario, the roadmap traces the cascade from initiating failure mechanisms, through their physical and electrical effects, to targeted material- or system-level solutions. The following sections provide a detailed analysis of these failure pathways and evaluate the efficacy of the proposed enhancement strategies.

### 6.1. Sweat Corrosion and Moisture Failure

In practical wearable applications, continuous perspiration introduces a humid, ion-rich microenvironment that severely challenges the long-term stability of skin-mounted electronics. Metallic conductors and 2D nanomaterials are inherently susceptible to this environment, where exposure to moisture, heat, and ambient air promotes rapid oxidation and galvanic corrosion. Consequently, unprotected sensing networks can undergo irreversible structural collapse and signal drift upon direct contact with sweat droplets [174]. Furthermore, hydrophilic substrates and embedded biomaterials degrade rapidly in aqueous environments, compromising the dynamic operational stability of wearable systems.

**Figure 11:**
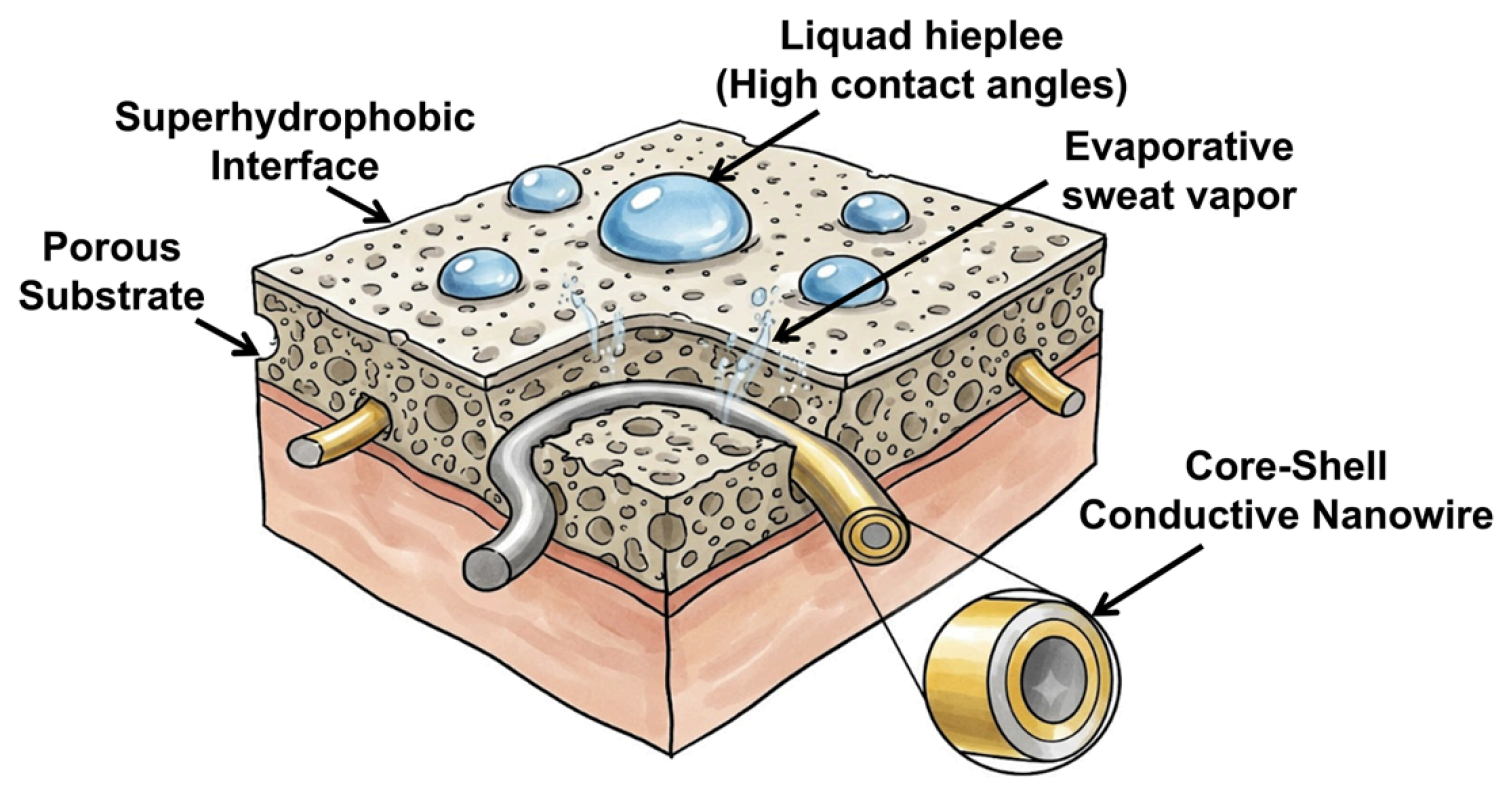
Moisture-resistant architecture based on core–shell nanowires and a superhydrophobic porous substrate.

To fundamentally address moisture-induced degradation without compromising intrinsic flexibility and breathability, researchers have developed advanced protection strategies focusing on core–shell nanoarchitectures and superhydrophobic permeable interfaces (Figure 11). At the nanoscale, core–shell encapsulation effectively isolates highly reactive metallic cores from oxidative environments while maintaining efficient electron transport. For example, coating vulnerable nanowires with noble metal shells provides robust protection against oxidation in high-humidity physiological solutions, fully preserving the conductor’s baseline performance [175]. This paradigm demonstrates superior oxidation and thermal stability compared to bare nanowires, concurrently suppressing cytotoxic ion leaching and broadening electrochemical stability windows. At the macroscopic level, complementary strategies focus on engineering superhydrophobic yet breathable interfaces that block liquid water infiltration while preserving unrestricted sweat vapor transport. Hierarchically porous films and superhydrophobic coatings act as dynamic moisture barriers, demonstrating stable operation across wet and underwater conditions [176,177]. Ultimately, synergizing nanoscale core–shell passivation with microscale superhydrophobic architectures enables breathable electronics to achieve a critical balance between liquid repellency, vapor permeability, and long-term reliability.

### 6.2. Mechanical Fatigue, Wear, and Delamination

Continuous body movements inflict cyclic multiaxial strains, shearing, and rubbing on skin-mounted electronics. This mechanical abuse drives microcrack propagation within rigid conductive networks and triggers delamination at modulus-mismatched soft–hard interfaces, inevitably culminating in catastrophic resistance spikes and open-circuit failures during long-term operation [178,179,180].

**Figure 12:**
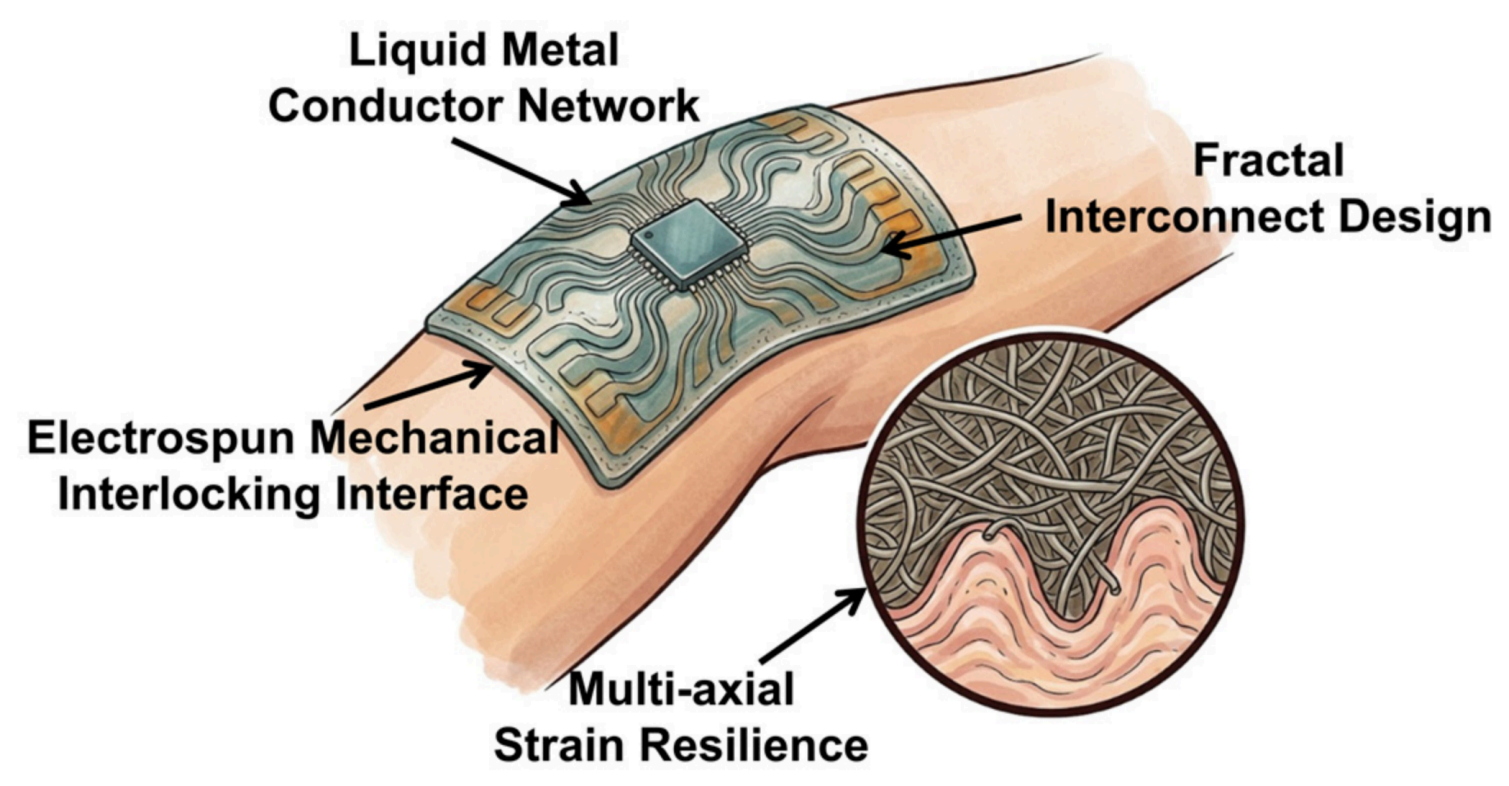
Anti-fatigue design utilizing LM networks and mechanical interlocking interfaces.

To bypass these degradation pathways, the design paradigm for breathable conductors must shift from static composites toward dynamic, stress-dissipating architectures. The functional role of this architecture is detailed in Figure 12, which illustrates how a LM conductor network synergizes with an electrospun mechanical interlocking interface. First, at the material level, rigid conductive fillers should be replaced by intrinsically deformable alternatives like gallium-based LMs, thereby eliminating particle embrittlement. Integrating these fluidic conductors with dynamic reversible polymer matrices (e.g., metal-ligand or hydrogen-bonded networks) establishes an essential autonomous self-healing mechanism, enabling the system to repair internal microcracks at room temperature [181,182]. Second, at the structural level, interfacial delamination must be managed through topological engineering rather than mere adhesive optimization [183]. Future designs should abandon traditional vulnerable planar substrates in favor of fibrous, mechanically interlocked architectures, such as electrospun nanofiber arrays or woven yarns [184]. These 3D porous topologies not only preserve essential breathability but intrinsically delocalize peak strains and blunt crack tips. Ultimately, combining dynamic self-healing chemistry with structurally interlocked topologies provides the definitive roadmap for ensuring the long-term electromechanical durability of breathable electronics.

### 6.3. Washing Damage and Anti-Fouling Strategies

For textile-integrated breathable electronics, long-term wear and routine laundering introduce severe mechanical and biochemical stressors. Standard household washing subjects devices to rotational shear, detergent chemistry, and thermal fluctuations, which aggressively induce conductor delamination, interfacial microcracks, and catastrophic circuit failure [185,186]. Simultaneously, prolonged epidermal contact fosters the accumulation of sweat, sebum, and cellular debris, promoting microbial biofilm colonization [187]. This biofouling not only clogs essential porous microclimates but also generates corrosive metabolic byproducts and interfacial electrical barriers that severely distort signal fidelity [188].

**Figure 13:**
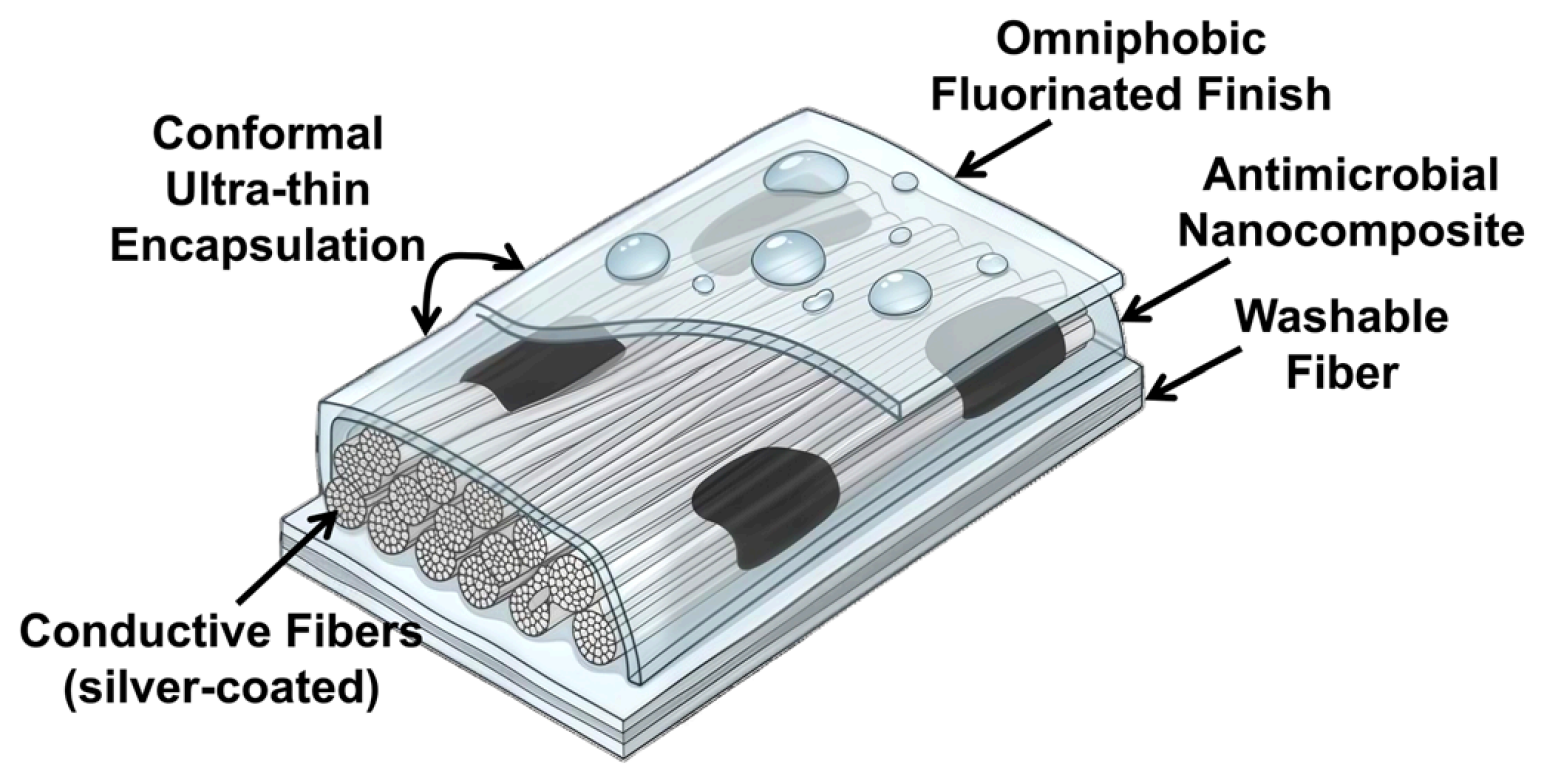
Washable and anti-fouling e-textile architecture with multi-layered encapsulation.

To ensure operational longevity, future e-textile designs must transcend passive substrates and adopt proactive interfacial and surface engineering. First, to withstand extreme laundering shear, researchers must prioritize strong covalent interfacial bonding, such as polydopamine cross-linking or in situ lamination, to irreversibly anchor conductive layers to textile fibers, fundamentally preventing shedding [189,190]. Second, conformal ultrathin encapsulation (e.g., parylene or TPU) should be applied specifically to fragile interconnections, shielding circuits from detergent liquid penetration while preserving intrinsic fabric breathability [65,191]. Finally, to combat bio-contamination, the integration of omniphobic fluorinated finishes alongside broad-spectrum antimicrobial nanocomposites (e.g., silver-polypyrrole) is imperative [189,192]. Figure 13 illustrates this synergistic, multi-layered defense: conformal encapsulation, omniphobic finishes, and antimicrobial nanocomposites collectively repel dirt, disrupt bacteria, and protect conductive fibers, thereby ensuring long-term structural and electrical stability.

### 6.4. Environmental Aging and Weatherability

Beyond epidermal interactions, breathable electronics face severe environmental aging from solar irradiance, thermal fluctuations, and oxidative atmospheres. Prolonged ultraviolet (UV) and radiant heat exposure significantly elevates device temperatures, accelerating the photo-oxidative degradation of polymer backbones and inducing premature strain failure in porous networks. Furthermore, unprotected photoactive nanomaterials can exacerbate this degradation by generating destructive oxygen radicals that aggressively attack the elastic host matrix [193,194,195].

**Figure 14:**
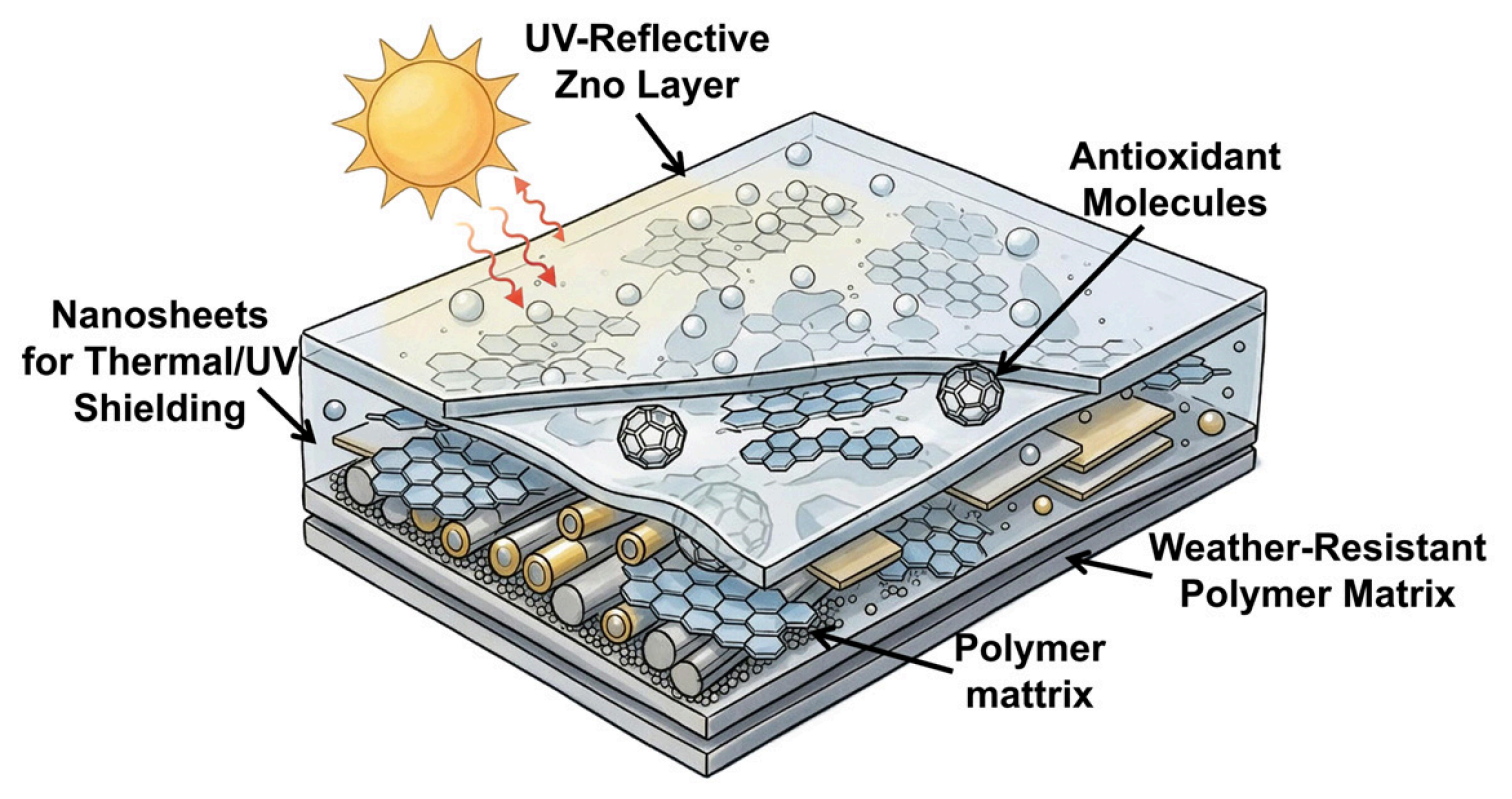
Environmentally resilient breathable structure with thermal/UV shielding and antioxidant protection.

To ensure long-term outdoor reliability, future designs must integrate intrinsic weatherability through targeted nanocomposite engineering and passivated architectures (Figure 14). First, to mitigate photo-oxidative decay, researchers should incorporate broad-spectrum UV-attenuating nanofillers, such as plasmonic Ag/BN, TiO_2_, or ZnO nanoparticles, into fibrous matrices like SEBS, TPU, or polyacrylonitrile. These additives effectively shield the polymer via photon absorption and electron-hole separation, while specific antioxidant blends can proactively scavenge destructive chemical radicals [195,196,197]. Second, thermal resilience must be reinforced by deploying high-temperature polymer backbones, such as radiative-cooling PDMS or highly robust polyimide aerogels capable of withstanding extreme temperatures (e.g., 460 °C) without compromising essential gas transport [193,198]. Finally, to protect internal conductive pathways from severe environmental oxidation, integrating core–shell metallic shielding (e.g., ultrathin Ag-Au networks) alongside shape-memory self-healing polymers provides a definitive roadmap for preserving both mechanical elasticity and electromechanical integrity during harsh environmental aging [71,199].

### 6.5. System Distortion and Algorithmic Compensation

Even with robust physical protection, breathable wearable sensors inevitably suffer from progressive material aging, viscoelastic hysteresis, and persistent baseline drift during long-term deployment. Furthermore, dynamic body movements introduce severe motion artifacts that physically deform transducer membranes, heavily distorting real-time physiological signals [200,201,202].

**Figure 15:**
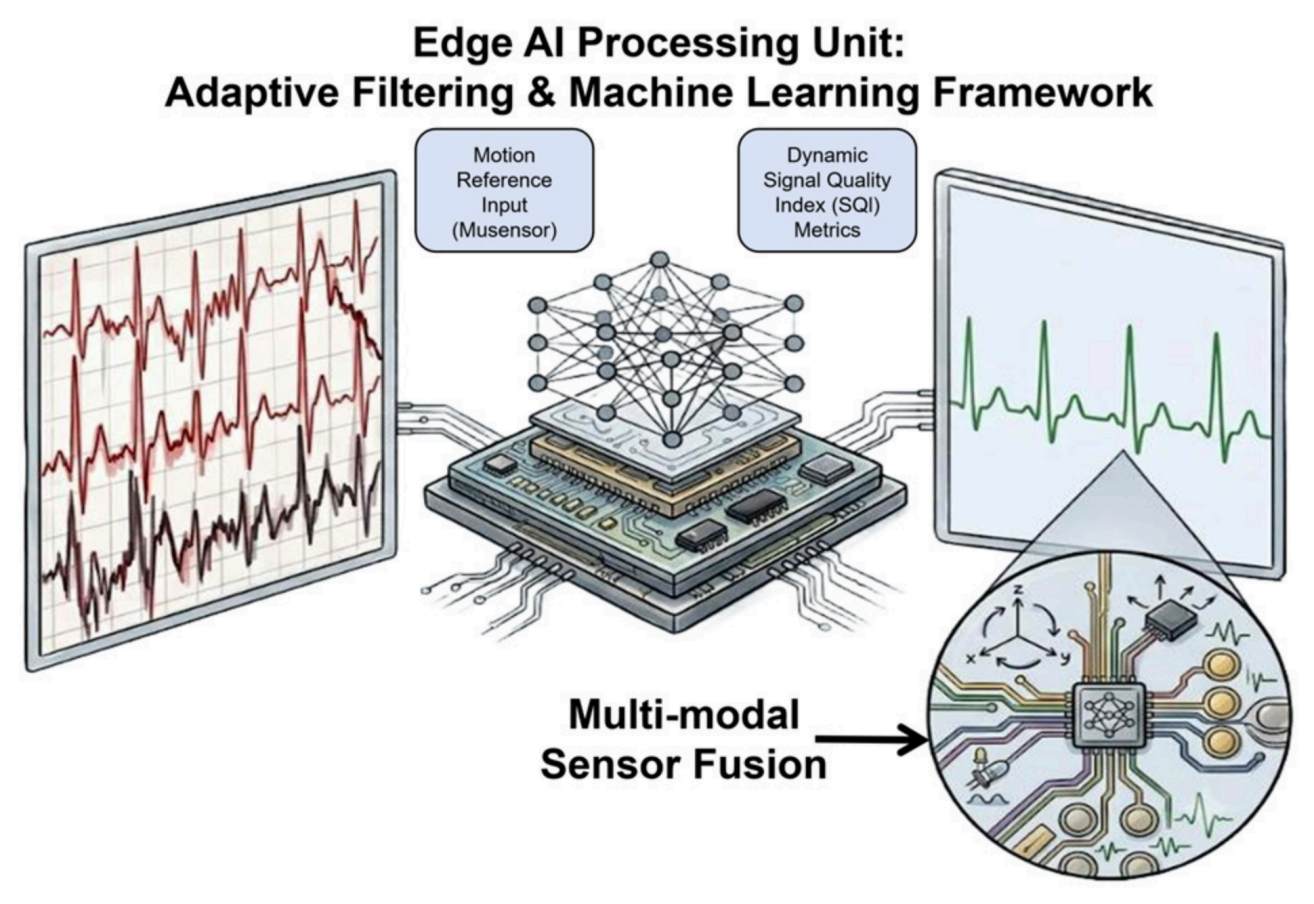
Edge AI processing unit for signal reconstruction via multi-modal fusion and adaptive filtering.

When physical hardware reaches its operational limits, advanced algorithmic compensation becomes essential. To counteract intrinsic material decay, future systems must deploy adaptive digital filtering and machine learning frameworks, such as autoregressive models or compact neural networks, to continuously calibrate baseline drift and non-linear hysteresis (Figure 15) [203]. Additionally, to resolve complex external motion artifacts, multi-modal sensor fusion, such as synchronizing IMU kinematics with primary biosignals, is critical for isolating gross physical interference [204,205]. Finally, implementing dynamic Signal quality index metrics allows systems to autonomously discard corrupted data [206]. Embedding these lightweight algorithms provides a definitive software-hardware co-design roadmap, ensuring high-fidelity signal acquisition despite inevitable physical degradation.

## 7. Conclusions

Breathable electronics have emerged as a transformative paradigm in flexible wearables, fundamentally resolving the “skin occlusion effect” and enabling continuous, high-fidelity physiological monitoring without causing skin inflammation or signal drift. By reconstructing conventional dense elastomers with intrinsically permeable architectures, these devices offer unique capabilities, including conformable adhesion, efficient thermal-moisture dissipation, and multi-modal sensing.

Throughout this review, we have systematically discussed the hierarchical design strategies, encompassing stretchable substrates, breathable microstructures, and conductive modifications, alongside discrete functional components and system-level integration formats like textiles and epidermal patches. Despite remarkable progress, significant technical bottlenecks continue to constrain practical deployment. Specifically, the electrochemical corrosion of nanomaterials, mechanical fatigue under dynamic wear, and the profound modulus mismatch between soft organic components and traditional rigid integrated circuits pose severe integration challenges. Furthermore, the lack of unified and standardized characterization protocols for evaluating breathability hinders comprehensive comparative analysis and commercialization.

Addressing these interconnected challenges will require holistic, interdisciplinary optimization. Future development must transition toward highly integrated, multifunctional platforms that can effectively suppress signal crosstalk during prolonged wear. Additionally, advancing on-device intelligence through smart algorithms will be crucial to balance real-time physiological data processing with stringent energy constraints. With ongoing synergistic advancements across materials science, integrated circuit manufacturing, and algorithm design, breathable electronics are expected to play a pivotal role in the next generation of unobtrusive, clinical-grade wearable health systems.

## Data Availability

No new original research data were created or analyzed in this review article. Therefore, data sharing is not applicable to this study. The review is based on previously published literature, and the relevant data supporting the discussed findings can be found in the original cited references.

## Abbreviations

The following abbreviations are used in this manuscript:

Abbreviations	Full description
2D	Two-dimensional
3D	Three-dimensional
AAO	Anodic aluminum oxide
BLE	Bluetooth Low Energy
BTMO	Binary transition metal oxide
BMO	Binary metal oxide
ECG	Electrocardiographic
ECoG	Electrocorticography
e-ECM	electronic extracellular-matrix
EEG	Electroencephalographic
EMG	Electromyographic
EOG	Electro-oculographic
e-skin	electronic skin
FEP	Fluorinated ethylene propylene
FGA	Flexible GelMA-aerogel
GF	Gauge factor
HMI	Human–machine interaction
ICP	Intrinsically conductive polymer
ITO	Indium tin oxide
LATP	Lithium aluminum titanium phosphate
L-EES	Large-area epidermal electronic system
LIG	Laser-induced graphene
LM	Liquid metal
LoRa	Long Range
MOF	Metal–organic framework
MUAP	Motor-unit action potential
NFC	Near-field communication
OLED	Organic light-emitting diode
PAN	Polyacrylonitrile
PA66	Polyamide 66
PDMS	Polydimethylsiloxane
PEG	Polyethylene glycol
PI	Polyimide
PLGA	Poly(lactic-co-glycolic acid)
PU	Polyurethane
PVA	Poly(vinyl alcohol)
PVD	Physical vapor deposition
PVDF-TrFE	Poly(vinylidenefluoride-trifluoroethylene)
R2R	Roll-to-roll
RFID	Radio-frequency identification
RH	Relative Humidity
SEBS	Styrene-ethylene-butylene-styrene
SEI	Solid electrolyte interphase
SEM	Scanning Electron Microscope
SLM	Semi-liquid metal
SNR	Signal-to-noise ratio
TENG	Triboelectric nanogenerator
TPE	Thermoplastic elastomer
TPU	Thermoplastic polyurethane
TSE	Thermosetting elastomer
UV	Ultraviolet
VDWTF	Van der Waals film
WTP	Water-assisted Transfer
WVP	Water Vapor Permeability
WVPR	Water Vapor Permeability Rate
WVTR	Water Vapor Transmission Rate
